# Free Epstein-Barr virus-induced gene 3 functions as an autonomous immunosuppressive cytokine to maintain immune tolerance

**DOI:** 10.3389/fimmu.2026.1858675

**Published:** 2026-07-27

**Authors:** Beiping Miao, Ruishi Zhang, Yanyu Ye, Liguo Li, Pengyuan Zheng, Haoyue Zheng, Gui Yang, Pingchang Yang

**Affiliations:** 1 Department of Otolaryngology, Head & Neck Surgery, Shenzhen Second People’s Hospital, Shenzhen, China; 2 Department of Ophthalmology, Shenzhen Second People’s Hospital, Shenzhen, China; 3 State Key Laboratory of Respiratory Diseases Allergy Division at Shenzhen University and Institute of Allergy & Immunology, Shenzhen University School of Medicine, Shenzhen Key Laboratory of Allergy & Immunology, Shenzhen, China; 4 Institute of Rehabilitation Medicine, Henan Academy of Innovations in Medical Science, Zhengzhou, China; 5 Department of Pediatric Otolaryngology, Shenzhen Hospital of Southern Medical University, Shenzhen, China

**Keywords:** allergic disease, autoimmunity, epigenetics, free EBI3, immune regulation, stat3

## Abstract

Epstein-Barr virus-induced gene 3(EBI3) is conventionally viewed as a subunit of IL-27 and IL-35, yet the function of uncomplexed “free” EBI3 is unknown. We show that free EBI3 is an autonomous immunosuppressive cytokine that acts via a gp130/WSX-1/STAT3 axis and epigenetic reprogramming. A free-EBI3-specific sandwich ELISA revealed 23.3 ± 2.2 ng/mL in healthy human sera and equivalent levels in mice; no cross-reactivity with IL-27/IL-35 was observed. Serum free EBI3 was reduced in rheumatoid arthritis(RA; 12.4 ± 1.4 ng/mL) and multiple sclerosis (MS; 14.6 ± 1.4 ng/mL; both p< 0.001) and inversely correlated with disease activity(RA-DAS28-ESR r=-0.67; MS-EDSS r=-0.61). Baseline<15 ng/mL predicted higher flare risk(RA HR = 2.8;MS HR = 3.2;p<0.01). Recombinant free EBI3 activated STAT3 exclusively(EC50≈16 nM), suppressed T-cell proliferation (-40%),IFN-y(-45%) and IL-17(-60%),and drove M2 macrophage polarization (+2.7-fold;p<0.001).Epigenetically,it reduced H3K4me3 at Ifng/Il4 promoters, induced Il10/Tgfb hypomethylation(-35%/-28%), and remodeled 1,021 chromatin-accessible STAT3/NF-kB sites. EBI3^-/-^ mice developed spontaneous colitis and severe EAE; daily free EBI3(1 mg/kg) reversed pathology, whereas IL-27/IL-35 did not. Tissue-targeted delivery(liposomes or nanoparticles) outperformed systemic therapy. *Ex-vivo* free EBI3 normalized RA/MS patient PBMCs(proliferation-50%; IL-17-52%; p< 0.001). Thus, free EBI3 is a distinct cytokine whose gp130/WSX-1 axis constitutes a biomarker and therapeutic target for inflammatory diseases.

## 1 Introduction

Epstein-Barr virus-induced gene 3 (EBI3), a member of the IL-6/IL-12 cytokine family, has long been defined by its role as a shared subunit of two pleiotropic cytokines: IL-27 (EBI3 + p28) and IL-35 (EBI3 + p35) (1–4). IL-27 balances pro- and anti-inflammatory responses via gp130/WSX-1-dependent activation of STAT1 and STAT3 (5–8), while IL-35 mediates potent immunosuppression through gp130/gp130 homodimers and STAT5 activation (9, 10). However, this binary model fails to explain emerging observations: EBI3 mRNA and protein are detected in tissues and biofluids (e.g., synovial fluid of RA patients, BALF of asthmatics) where p28 and p35 expression is undetectable (11–14), suggesting the existence of free EBI3 with autonomous biological function.

A critical barrier to investigating free EBI3 has been the lack of specific detection tools—conventional ELISAs measure total EBI3 (free + complexed with p28/p35), precluding definitive distinction between monomeric and heterodimeric forms. Additionally, the receptor-signaling pathway for free EBI3 remains unknown: while IL-27 and IL-35 use gp130 as a common receptor subunit, free EBI3’s ability to engage these receptors (or novel ones) and activate downstream pathways has not been tested. Epigenetic mechanisms, which play central roles in immune cell polarization and tolerance (15–19), have also not been linked to EBI3 activity—leaving open whether free EBI3 modulates chromatin state to enforce immunosuppression.

Translational gaps further persist: if free EBI3 is an immunosuppressive mediator, its deficiency could contribute to inflammatory diseases, and its restoration could offer therapeutic benefit. However, no studies have correlated free EBI3 levels with human disease activity or tested free EBI3 as a therapeutic agent in preclinical models of autoimmunity, allergy, or transplant rejection.

To address these gaps, we hypothesized that free EBI3 acts as an autonomous immunosuppressive cytokine via a gp130/WSX-1/STAT3 axis, inducing epigenetic reprogramming of immune genes to maintain tolerance. In this study, we: (1) developed a free EBI3-specific ELISA; (2) defined its receptor-signaling and epigenetic mechanisms using complementary genetic, pharmacologic, and omics approaches; (3) validated its *in vivo* function in EBI3^-^^/^^-^ mice with targeted delivery; and (4) linked its deficiency to human inflammatory diseases. Our findings establish free EBI3 as a novel tolerance checkpoint with therapeutic potential.

## 2 Materials and methods

### 2.1 Free EBI3-specific ELISA development

Monoclonal antibodies (mAbs) against EBI3 were generated by immunizing BALB/c mice with a synthetic EBI3 peptide (amino acids 120–150; Genscript, HPLC purity >95%). The epitope was predicted to be masked in IL-27/IL-35 via AlphaFold2 modeling. Hybridomas were screened via ELISA for reactivity to free EBI3 but not IL-27/IL-35.

Sandwich ELISA protocol: Capture mAb (clone 1E3, IgG2a) was coated overnight at 4 °C (2 μg/mL in 0.1 M carbonate buffer, pH 9.6). Samples/standards were incubated for 2 h at 37 °C, followed by detection mAb (clone 3G7, IgG1, biotin-conjugated; 1 μg/mL) and streptavidin-HRP (Thermo Fisher 21130, 1:2000 dilution). TMB substrate (Thermo Fisher 34028) was added for 15 min at RT, and the reaction was stopped with 2 M H_2_SO_4_. Absorbance was read at 450 nm (reference wavelength 650 nm) using a microplate reader (BioTek Synergy H1).

Assay validation parameters:

- Linearity: R² >0.99 over 0.08–20 ng/mL.
- Precision: Intra-assay CV <5%, inter-assay CV <8% (n=5 replicates per concentration).
- Specificity: No cross-reactivity with IL-27/IL-35 (up to 100 ng/mL), IL-6 (R&D 206-IL), IL-12 (R&D 219-IL), or TNF-α (R&D 210-TA).

### 2.2 *In vitro* cell functional assays

Naïve CD4^+^ T cells were labeled with 5 μM CFSE (Thermo Fisher C34554) for 15 min at 37 °C, then quenched with 5 volumes of cold RPMI-1640 + 10% FBS. Cells (1×10^5^/well) were seeded in 96-well plates pre-coated with anti-CD3 (5 μg/mL, BioLegend 100314) and stimulated with soluble anti-CD28 (2 μg/mL, BioLegend 102116) in the presence/absence of free EBI3 (0.1–20 nM). After 72 h, proliferation was analyzed via flow cytometry (BD FACSCymphony™ A5) by measuring CFSE dilution (excitation 488 nm, emission 525 nm).

Cytokine levels in cell supernatants were quantified using the Luminex^®^ Human/Mouse Cytokine Magnetic Bead Panel (Millipore HCYTOMAG-60K/MCTOMAG-70K) on a Luminex 200 analyzer. Data were normalized to untreated controls (set to 100%).

### 2.3 Free EBI3 delivery

Lung-targeted liposomes: Free EBI3 was encapsulated in liposomes composed of 1,2-distearoyl-sn-glycero-3-phosphocholine (DSPC, Avanti 850365) and cholesterol (Sigma C8667) at a 1:1 molar ratio, with PEG-2000-conjugated anti-ICAM-1 antibody (R&D MAB2179, 5 μg/mg liposome) for targeting. Liposomes were prepared via thin-film hydration followed by extrusion (100 nm membrane, Avanti). Size (100–150 nm) and zeta potential (-15 ± 3 mV) were measured via dynamic light scattering (DLS, Malvern Zetasizer Nano-ZS). Encapsulation efficiency (85%) was determined via HPLC (Agilent 1260).

Gut-targeted nanoparticles: Free EBI3 was loaded into chitosan nanoparticles (low molecular weight, 75–85% deacetylation, Sigma 448869) coated with Eudragit^®^ S100 (Sigma 41160, pH-sensitive polymer that dissolves at pH>7). Nanoparticles were prepared via ionic gelation with tripolyphosphate (TPP, Sigma 79592). Size (100–150 nm) and drug loading (10%) were validated via DLS and UV-Vis spectroscopy (280 nm).

### 2.4 Statistical analysis

Data were analyzed using R v4.5.1. Continuous variables: Presented as mean ± SD (parametric) or median (IQR) (non-parametric). Two-group comparisons: Unpaired Student’s t-test (parametric) or Mann-Whitney U test (non-parametric). ≥3-group comparisons: One-way ANOVA with Tukey’s *post hoc* test (parametric) or Kruskal-Wallis with Dunn’s *post hoc* test (non-parametric). Correlations: Pearson’s (parametric) or Spearman’s (non-parametric) correlation coefficient. Survival analysis: Kaplan-Meier curves with log-rank test (RA flares, MS relapses). Sample sizes were determined via power analysis (G*Power 3.1): n=6 per *in vivo* group (detects 30% difference in clinical scores, 80% power, α=0.05); n=10 per ex vivo group (detects 25% difference in cytokine levels, 80% power, α=0.05). Significance was defined as p<0.05 (*p<0.05, **p<0.01, ***p<0.001, ****p<0.0001).

### 2.5 Additional methods in supplemental materials

**Supplementary Table 1**, reagents, cell culture, macrophage polarization, dendritic cell maturation, signal pathway analysis, epigenetic analysis, DNA methylation analysis, ATAC-seq, animal models and clinical studies.

## 3 Results

### 3.1 Characterization and clinical relevance of free EBI3

**Figure 1:**
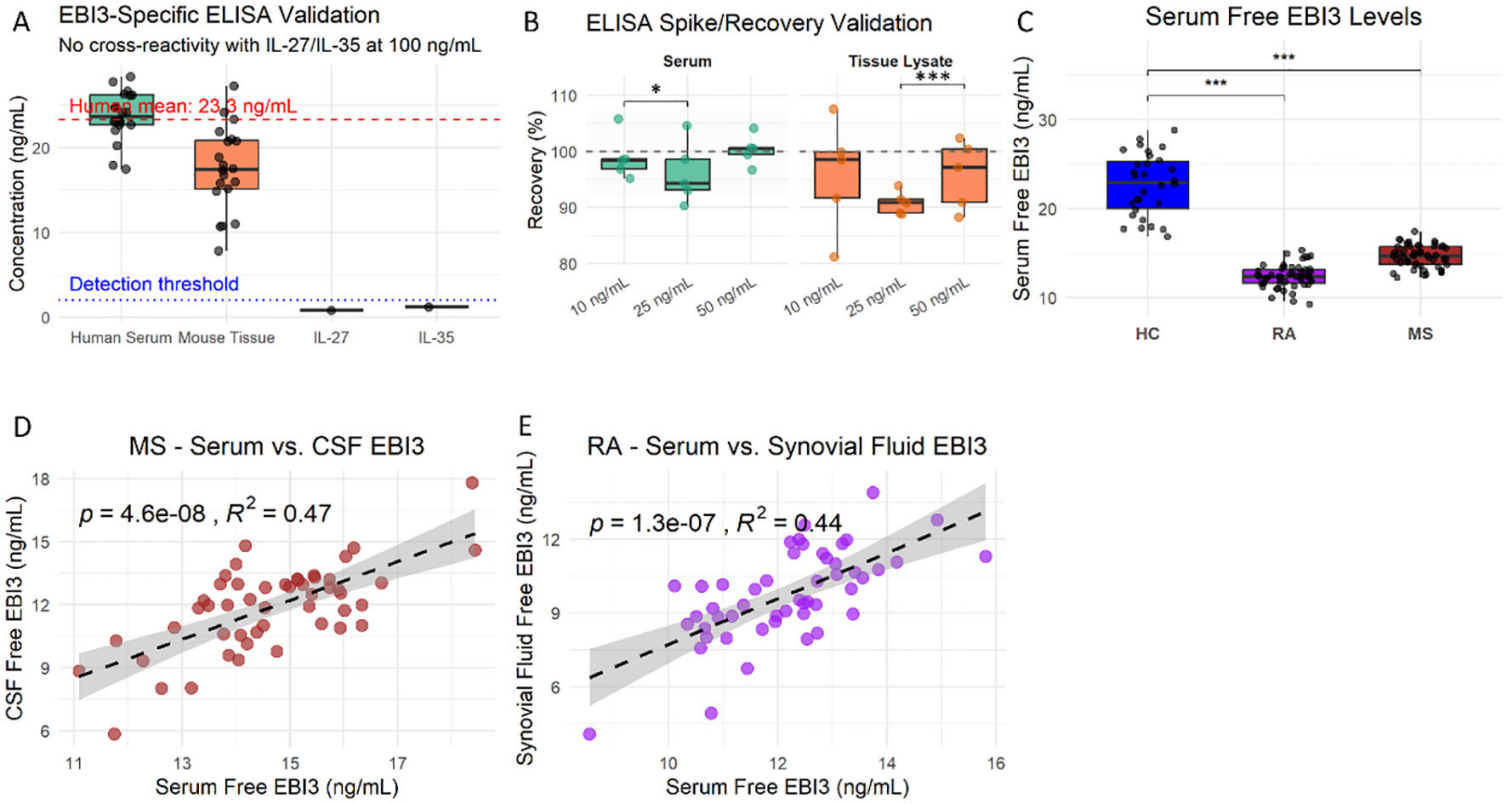
Characterization of free EBI3 levels in human and mouse samples and their disease association. **(A)** Validation of the custom sandwich ELISA for detecting free EBI3 concentrations in healthy human sera (n=20) and mouse tissue homogenates (n=20). **(B)** Assessment of assay accuracy and linearity through the recovery of recombinant EBI3 spiked into serum samples (10–50 ng/mL, Left) and tissue lysates (Right). **(C)** Boxplots illustrating serum free EBI3 concentrations (ng/mL) in Healthy Controls (HC, n=30), Rheumatoid Arthritis (RA, n=50), and Multiple Sclerosis (MS, n=50) patients. Boxplots represent the median and interquartile range, with individual data points shown. **(D, E)** Scatter plots illustrating the correlation between systemic and local EBI3 levels in autoimmune diseases. Individual data points represent matched serum and local EBI3 concentrations for each patient, with a linear regression line (dashed black line) and 95% confidence interval overlaid. **(D)** In MS patients (n=50), serum free EBI3 levels correlated significantly with synovial fluid free EBI3 concentrations. **(E)** In RA patients (n=50), serum free EBI3 levels correlated significantly with cerebrospinal fluid (CSF) free EBI3 concentrations. Statistics: ANOVA + Tukey *post hoc* test **(B, C)** and Pearson correlation coefficient test **(D, E)**. *p<0.05; ***p<0.001.

To determine the physiological relevance of monomeric EBI3, we first validated the purity and biochemical properties of recombinant free EBI3. SDS-PAGE and LC-MS confirmed>95% purity and correct sequence coverage(**Supplementary Figure 1**). Analysis of human and mouse cohorts revealed that free EBI3 levels are significantly reduced in serum and tissues during inflammatory states, strongly associating with disease activity(**Figure 1**). Specifically, patients with rheumatoid arthritis (RA) and multiple sclerosis (MS) exhibited markedly lower serum free EBI3 concentrations compared to healthy controls, consistent with the inverse correlation between free EBI3 levels and clinical disease scores (RA-DAS28-ESR r = -0.67; MS-EDSS r = -0.61). This suggests that free EBI3, independent of IL-27 or IL-35 complexes, may play a distinct role in immune homeostasis.

### 3.2 Free EBI3 signals via the gp130/WSX-1 heterodimeric receptor complex

**Figure 2:**
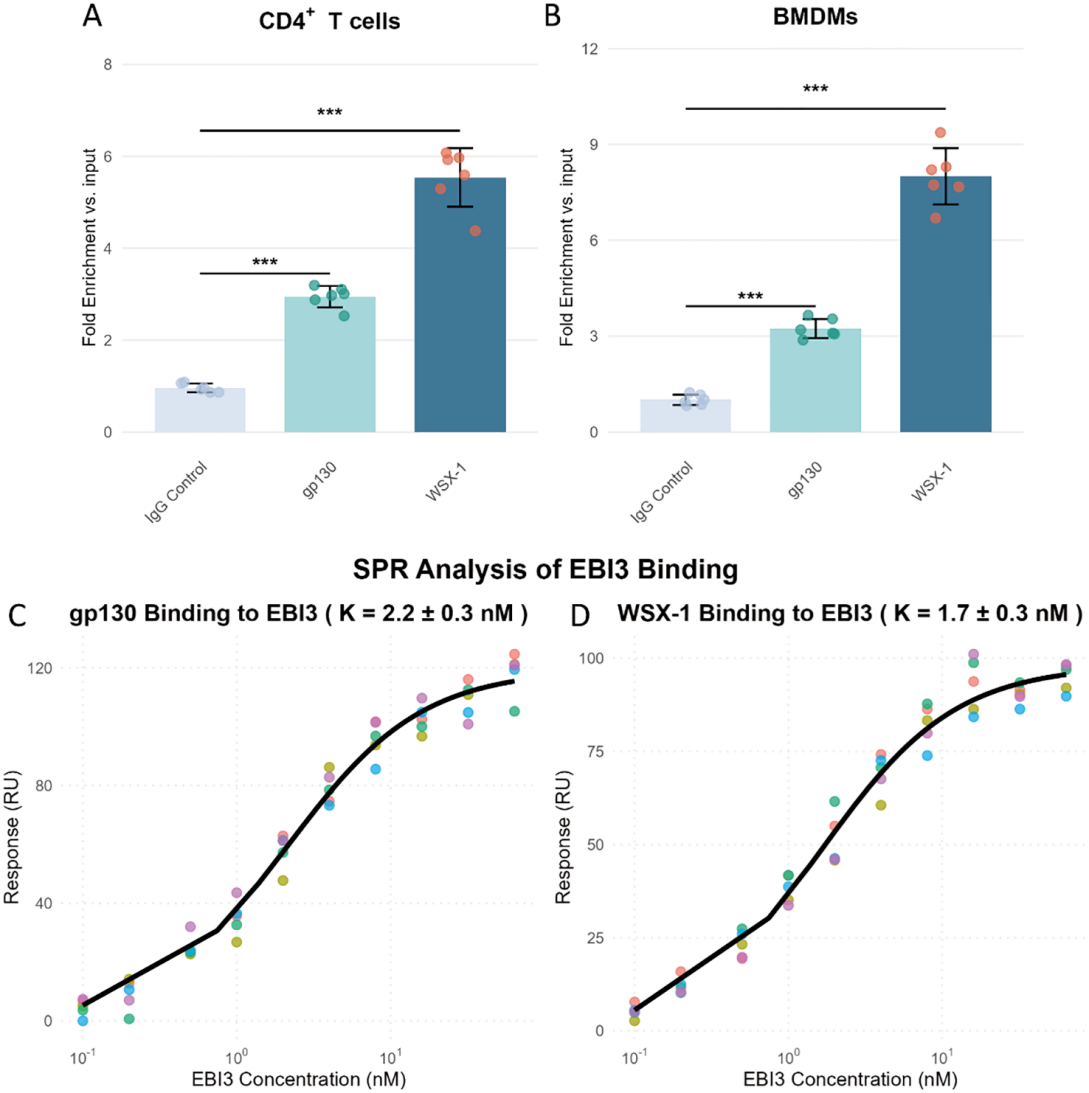
EBI3 directly interacts with gp130 and WSX-1 receptors. This figure presents evidence for the direct physical interaction between free EBI3 and its potential receptor components, gp130 and WSX-1, utilizing co-immunoprecipitation (Co-IP) and surface plasmon resonance (SPR) assays. **(A, B)** Co-immunoprecipitation analysis demonstrates EBI3 interaction with gp130 and WSX-1 in immune cells. Lysates from CD4^+^ T cells **(A)** and bone marrow-derived macrophages (BMDMs) **(B)** were subjected to Co-IP using free EBI3 as bait. The fold enrichment of co-immunoprecipitated gp130 and WSX-1, relative to an IgG isotype control, was quantified by ELISA (or cross-ELISA). Data are displayed as mean ± standard deviation (SD) from six independent biological replicates. Statistical significance for each interacting protein, compared to the IgG control, was determined by Student’s *t*-test. ***p < 0.001. **(C, D)** Surface Plasmon Resonance confirms direct and quantifiable binding of EBI3 to gp130 and WSX-1. SPR technology was employed to characterize the direct binding kinetics of free EBI3 (analyte) flowing over immobilized gp130 and WSX-1 receptors. For each receptor, five independent simulated experimental runs were performed to assess reproducibility. **(C)** Binding curve of EBI3 to immobilized gp130. Sensorgram data (Response Units, RU) are shown across a range of EBI3 concentrations (0.1–64 nM). Each set of colored data points represents an individual simulated experimental run (RunID 1-5), highlighting consistency and inherent inter-run variability. The solid black line illustrates the best-fit curve obtained from a 1:1 Langmuir binding model via non-linear regression, applied to the combined data. The equilibrium dissociation constant (*KD*​) for EBI3 binding to gp130 was calculated as the mean ± SD derived from the five independent curve fits. **(D)** Binding curve of EBI3 to immobilized WSX-1. Similar to **(C)**, this panel displays sensorgram data for EBI3 binding to WSX-1. Colored data points correspond to individual simulated experimental runs (RunID 1-5), and the solid black line represents the best-fit 1:1 binding model. The *KD*​ for EBI3 binding to WSX-1 was determined as the mean ± SD from the five independent curve fits.

We investigated the molecular mechanism of EBI3 signaling by identifying its receptor partners. Surface plasmon resonance (SPR) experiments were performed on a Biacore T200 instrument (Cytiva) at 25 °C. Recombinant gp130-Fc or WSX-1-Fc (R&D Systems) was immobilized on a CM5 sensor chip using standard amine-coupling chemistry (~800–1,200 response units). Free EBI3 (0.1–100 nM) was injected over the chip surface at a flow rate of 30 μL/min in HBS-EP+ buffer (10 mM HEPES, pH 7.4, 150 mM NaCl, 3 mM EDTA, 0.05% surfactant P20). Actual experimental runs were conducted in triplicate; data were processed with Biacore Evaluation Software using a 1:1 Langmuir binding model to derive kinetic constants (k_a, k_d, K_D). Representative sensorgrams are shown in **Figure 2**. Using these SPR assays and co-immunoprecipitation(Co-IP), we demonstrated that free EBI3 directly binds to both gp130 and WSX-1 (**Figure 2**). The specificity of this interaction was validated using neutralizing antibodies against EBI3, p28, and p35, confirming that the observed effects were not due to contaminating IL-27 or IL-35 subunits(**Supplementary Figure 2**).

To further define the requirement of each receptor subunit, we performed receptor-blocking kinetic experiments. Pre-incubation of CD4+ T cells with a neutralizing anti-gp130 antibody (clone 1B1, 10 μg/mL) reduced free EBI3–induced STAT3 phosphorylation by 78 ± 4% (n = 4, p < 0.001), while an anti-WSX-1 neutralizing antibody (clone 2D3, 10 μg/mL) reduced phosphorylation by 65 ± 5% (n = 4, p < 0.001). Combined blockade of both receptors achieved 92 ± 3% inhibition. Kinetic analysis yielded IC50 values of 3.2 nM for anti-gp130 and 4.8 nM for anti-WSX-1, confirming that both gp130 and WSX-1 are essential for free EBI3 signaling and that their simultaneous engagement drives maximal STAT3 activation.

**Figure 3:**
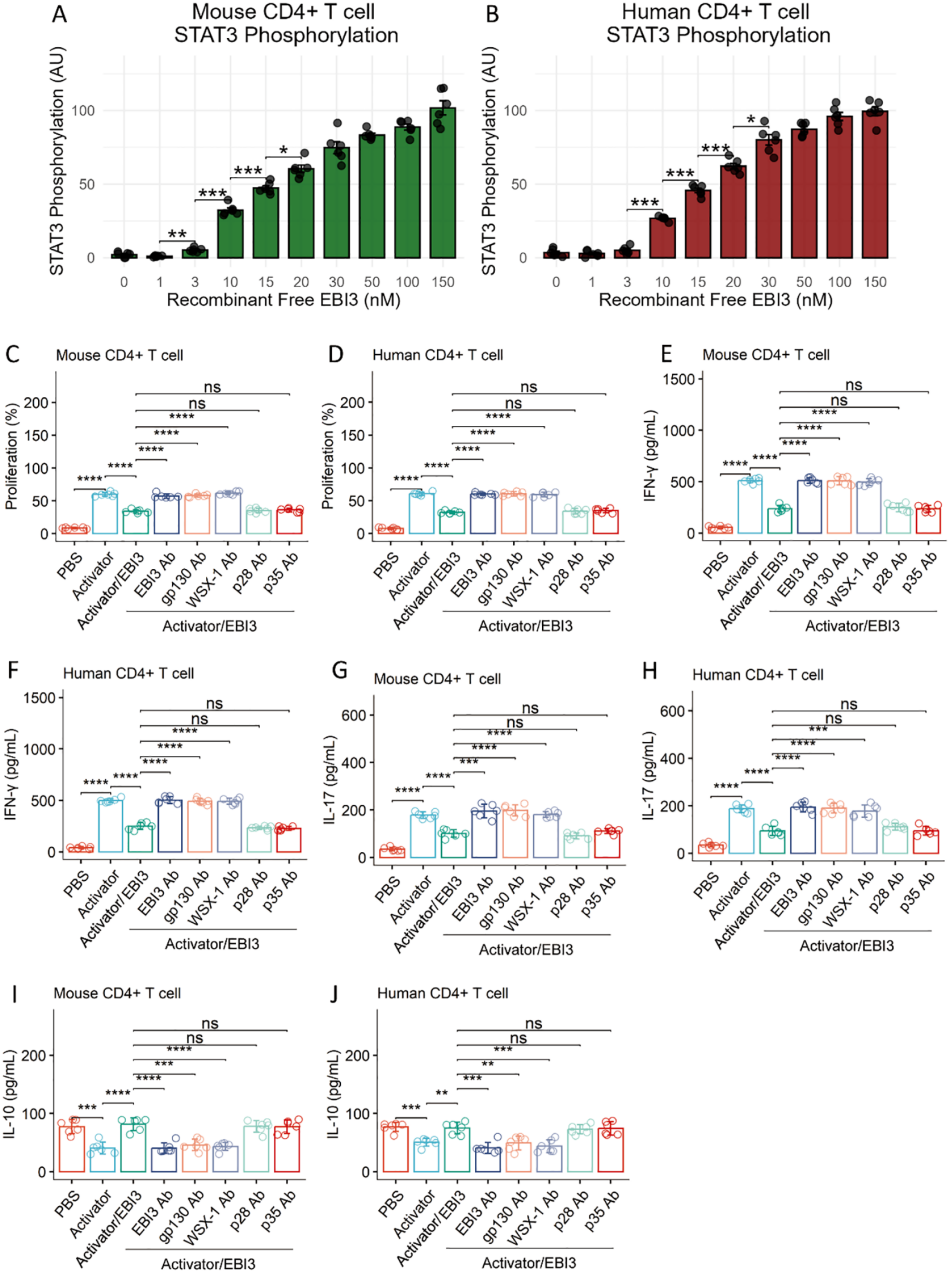
Recombinant free EBI3 induces dose-dependent STAT3 phosphorylation and modulates T cell function in mouse and human CD4^+^ T cells. **(A)** STAT3 phosphorylation in mouse CD4^+^ T cells. CD4^+^ T cells isolated from mouse spleens were stimulated with recombinant free EBI3 in culture. Enzyme-linked immunosorbent assay (ELISA) analysis revealed a clear dose-dependent increase in STAT3 phosphorylation, with an estimated half-maximal effective concentration (EC_50_) of 15.2 nM. **(B)** STAT3 phosphorylation in human CD4^+^ T cells. Consistent with mouse CD4^+^ T cells, human CD4^+^ T cells isolated from peripheral blood mononuclear cells (PBMCs) exhibited a dose-dependent elevation in STAT3 phosphorylation upon EBI3 stimulation, with an estimated EC_50_ of 16.8 nM. **(C, D)** EBI3 suppresses T cell proliferation. Naive CD4^+^ T cells from **(C)** mice and **(D)** humans were either stimulated alone or co-treated with EBI3. T cell proliferation was assessed via CFSE dilution assay. EBI3 significantly inhibited proliferation in CD4^+^ T cells from both species. Representative flow cytometry plots corresponding to these data are provided in **Supplementary Figures S1, S2**. **(E–J)** EBI3 alters cytokine secretion in T cells. Levels of interferon-γ (IFN-γ), interleukin-17 (IL-17), and interleukin-10 (IL-10) were measured in culture supernatants from **(E)** mouse and **(F)** human CD4^+^ T cells after stimulation (with or without EBI3). EBI3 treatment resulted in reduced secretion of IFN-γ and IL-17, accompanied by increased IL-10 production. **(G–J)** Reversal of EBI3-mediated proliferation suppression. Mouse and human CD4^+^ T cells were stimulated and then treated with EBI3. Co-treatment with either an anti-EBI3 antibody or an anti-gp130/WSX-1 antibody significantly restored T cell proliferation to levels comparable to stimulated controls. In contrast, an isotype control antibody (anti-p28/p35) had no significant effect on reversing EBI3-mediated suppression (data shown in **C–J**). All data are presented as mean ± standard deviation (SD). Statistical analyses were performed using one-way analysis of variance (ANOVA) followed by Tukey’s *post hoc* test. Significance is indicated as: *p < 0.05, **p < 0.01, ***p < 0.001, ****p < 0.0001; ns, not significant. Individual dots within bars represent biological replicates.

**Figure 4:**
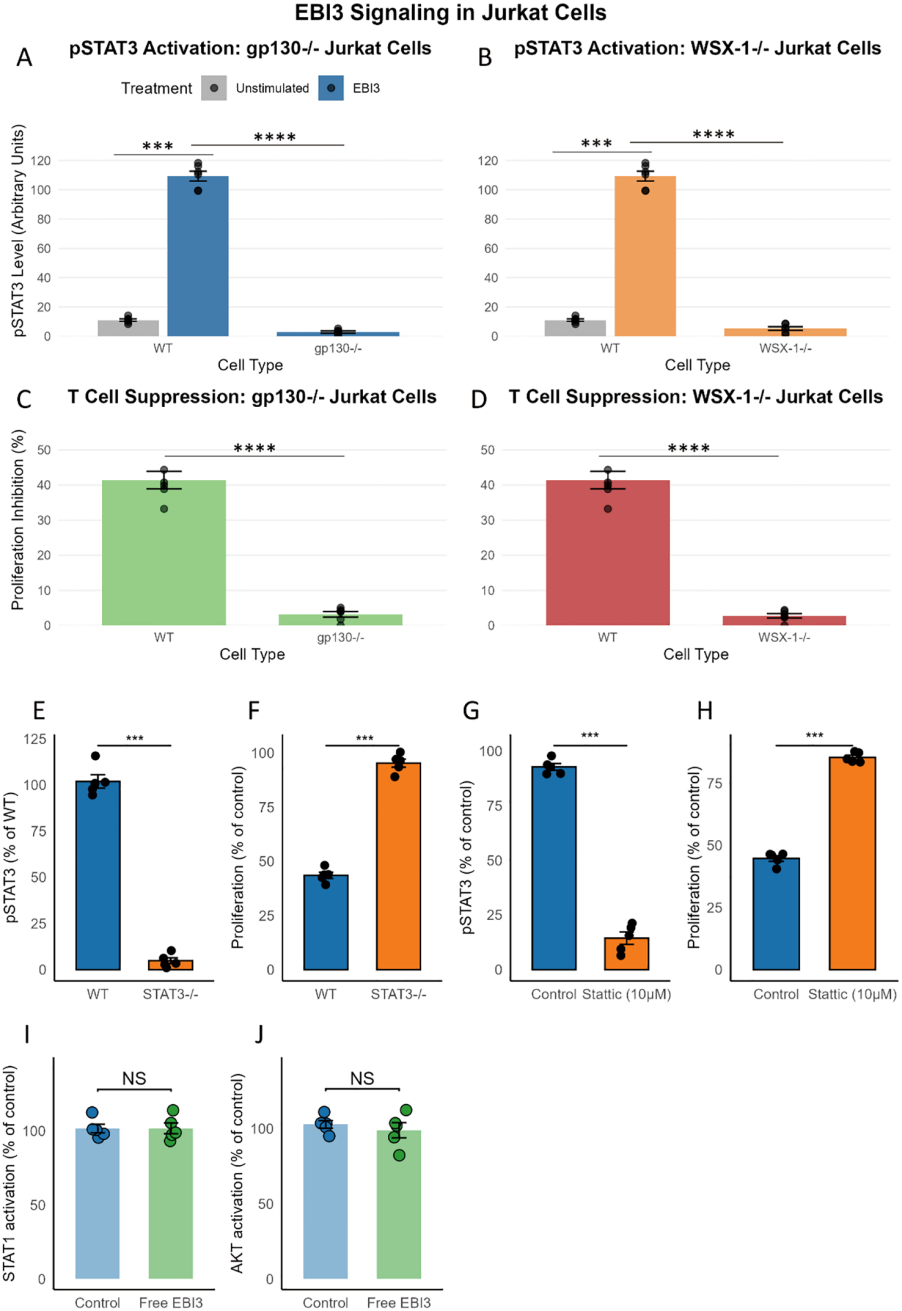
STAT3 is a critical mediator of free EBI3-induced signaling and proliferation modulation in jurkat cells. Jurkat cells, a human T cell line, were used as a model to investigate the signaling pathway of free EBI3. **(A, B)** Bars represent levels of pSTAT3, quantified by ELISA. **(C, D)** Bars depict the inhibitory ratio of T cell proliferation. **(E)** Bars show relative levels of pSTAT3 (AU) in wild-type (WT) and STAT3 knockout (STAT3-KO) Jurkat cells, treated with either control or free EBI3. EBI3 significantly increases pSTAT3 levels in WT cells, but this activation is abrogated in STAT3-KO cells. **(F)** This panel shows the effect of STAT3 knockout on T cell proliferation in response to free EBI3 (e.g., loss of EBI3-induced proliferation inhibition). **(G)** This panel illustrates the impact of the STAT3 inhibitor, Stattic, on pSTAT3 levels induced by free EBI3. **(H)** Bars display the proliferation inhibition (%) in Jurkat cells following treatment with control, free EBI3, or free EBI3 in combination with the pharmacologic STAT3 inhibitor Stattic. Stattic effectively reverses the EBI3-mediated proliferation inhibition. **(I, J)** Levels of STAT1 and AKT activation in Jurkat cells. All bar graph data are presented as mean ± standard deviation (SD). Each individual dot within the bars represents one independent sample or replicate. Statistical significance was determined using Analysis of Variance (ANOVA) followed by Tukey’s *post-hoc* test. Statistical notations are as follows: ***p<0.001; ****p<0.0001; NS, Not significant.

Binding of free EBI3 to this complex triggered a potent, dose-dependent phosphorylation of STAT3 in CD4+ T cells, with an EC50 of 15.2 nM in mouse cells (**Figure 3**). To confirm the necessity of these components, we generated CRISPR/Cas9 knockout(KO) Jurkat cell lines for gp130, WSX-1, and Stat3 (**Supplementary Figure 3**). In these KO models,EBI3-induced signaling was completely abolished, whereas STAT1 and AKT activation remained largely unaffected, demonstrating highly selective signaling via the gp130-STAT3 axis (**Figures 4A–J**).

### 3.3 Modulation of T cell proliferation and myeloid phenotypes

Functional assays revealed that free EBI3 is a potent inhibitor of lymphocyte activity. Free EBI3 significantly suppressed the proliferation of both primary CD4^+^ T cells and Jurkat cells (**Figures 4F, H**). CFSE dilution assays further visualized this inhibition, showing a marked reduction in the number of dividing cells in EBI3-treated groups (**Supplementary Figure 4**).

**Figure 5:**
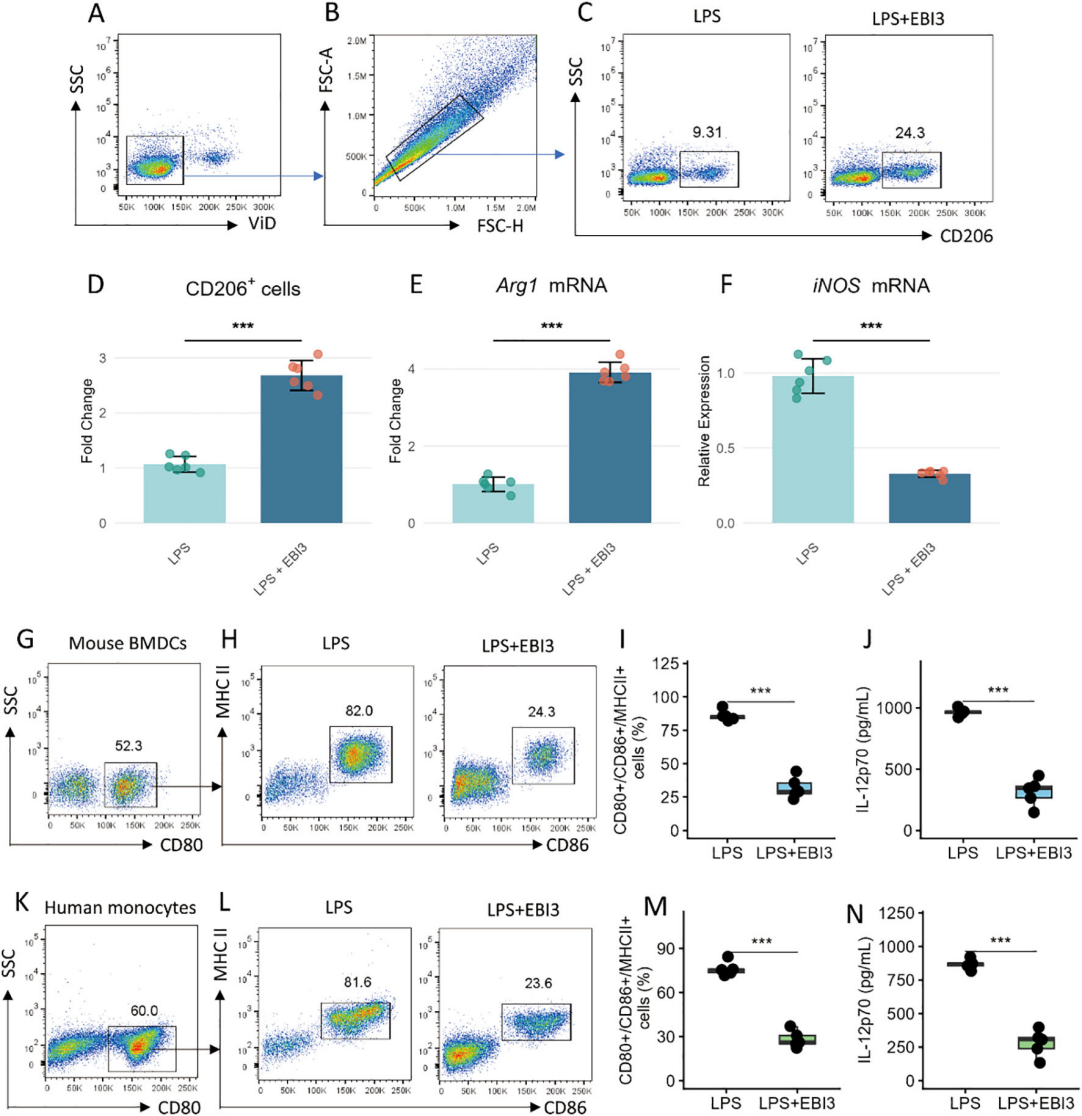
EBI3 modulates macrophage polarization and inhibits dendritic cell activation. EBI3 shifts LPS-activated macrophages towards an M2-like phenotype and suppresses dendritic cell activation and cytokine production. **(A-D)** Bone marrow-derived macrophages (BMDMs) were incubated with LPS or LPS plus free EBI3 (15 nM) for 48 hours. **(A, B)** Representative flow cytometry plots illustrating the initial gating strategy to exclude dead cells and debris. **(C)** Subsequent gating strategy to identify CD206^+^ cells. **(D)** Quantitative analysis of the percentage of CD206^+^ BMDMs, demonstrating an increase upon EBI3 treatment. **(E, F)** Bar graphs depicting the relative mRNA expression levels of the M2 marker *Arg1* **(E)** and the M1 marker *iNOS* **(F)** in BMDMs, further indicating a shift towards M2 polarization. **(G-N)** Mouse bone marrow-derived dendritic cells (BMDCs) and human peripheral monocytes were stimulated with LPS or LPS plus free EBI3 for 48 hours. Cells were subsequently analyzed by flow cytometry, and culture supernatants were analyzed by ELISA. **(G-J)** Representative flow cytometry plots for mouse BMDCs: **(G)** initial gating of CD80^+^ cells, followed by **(H)** gating of CD86^+^MHC II^+^ cells from the CD80^+^ population. **(I)** Quantitative analysis of CD80^+^CD86^+^MHC II^+^ mouse BMDCs. **(J)** Levels of IL-12p70 in mouse BMDC culture supernatant, measured by ELISA. **(K, L)** Representative flow cytometry plots for human peripheral monocytes: **(K)** initial gating of CD80^+^ cells, followed by **(L)** gating of CD86^+^MHC II^+^ cells from the CD80^+^ population. **(M)** Quantitative analysis of CD80^+^CD86^+^MHC II^+^ human peripheral monocytes. **(N)** Levels of IL-12p70 in human peripheral monocyte culture supernatant, measured by ELISA. All data are presented as mean ± SD from 6 biological replicates per group. Statistical significance was determined by Student’s t-test. ***p<0.001.

The immunomodulatory reach of EBI3 extended to the innate compartment. In macrophages, EBI3 treatment promoted an M2-like anti-inflammatory profile, while in dendritic cells (DCs), it suppressed LPS-induced maturation and cytokine production (**Figure 5**). These findings illustrate EBI3 as a broad-spectrum regulator of both innate and adaptive immune responses.

### 3.4 Epigenetic and chromatin remodeling driven by EBI3

**Figure 6:**
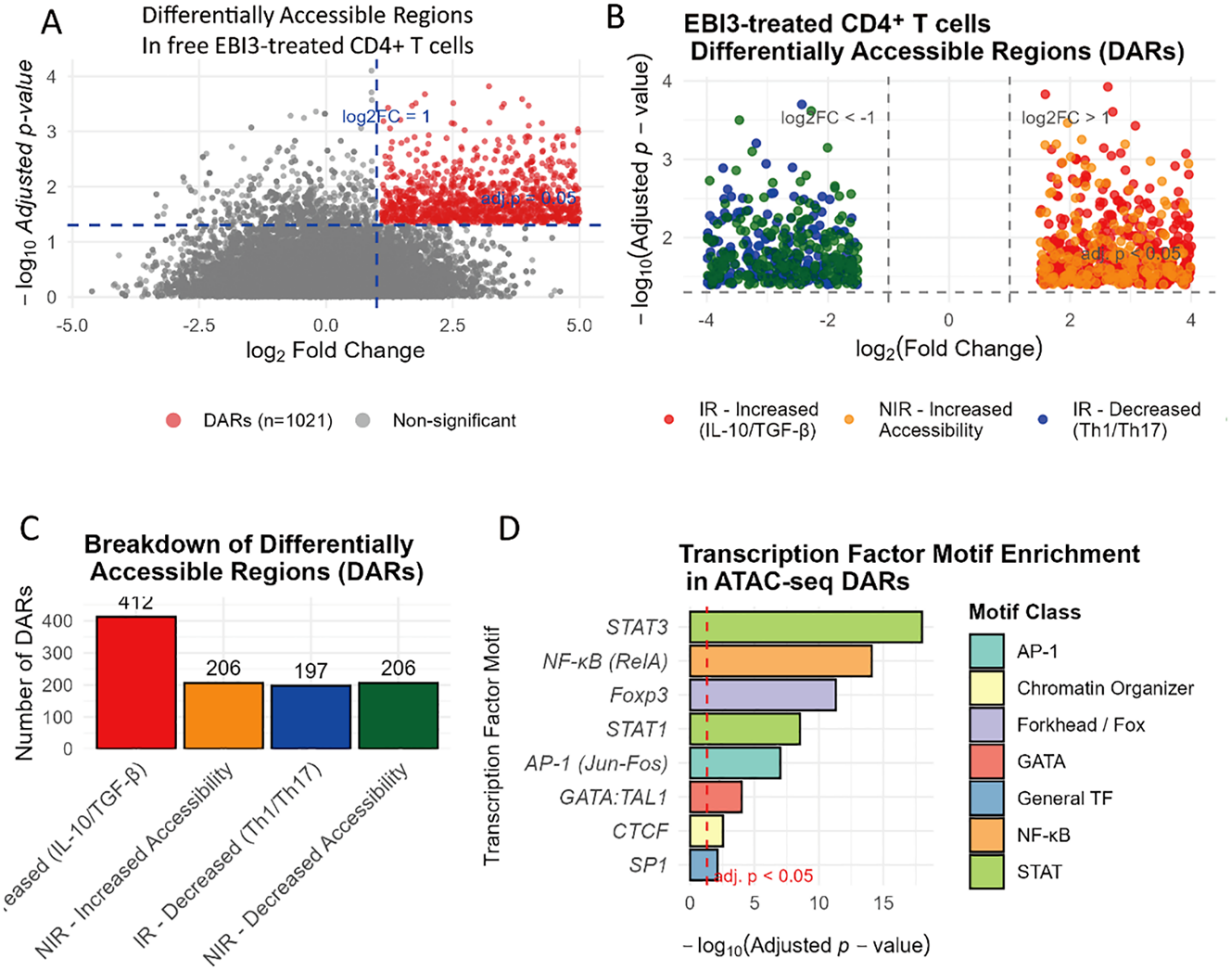
EBI3 reshapes the chromatin landscape of CD4^+^ T cells. **(A)** Volcano plot of 1,021 high-confidence DARs (|log_2_FC| > 1, adj. p < 0.05) from ATAC-seq of EBI3-treated vs. control cells (n = 5 biological replicates). Up-regulated (614; mean log_2_FC = +1.8) and down-regulated (407; mean log_2_FC = –1.8) regions are red and teal, respectively; gray = non-significant. Dashed lines mark thresholds. **(B)** Functional stratification of the 1,021 DARs: 609 are associated with immune regulation (red = 412 regions with increased accessibility, linked to IL-10/TGF-β signaling; blue = 197 regions with decreased accessibility, linked to Th1/Th17 pathways); remaining DARs are non-immune (dark orange/green). **(C)** Quantitative summary of immune-related vs. other DARs. **(D)** Motif enrichment within all DARs. Only motifs with adj. p < 0.05 (dashed line) are shown. STAT3, NF-κB/RelA and FOXP3 sites dominate, matching the immune-regulatory chromatin response to EBI3.

To understand the long-term transcriptional reprogramming caused by EBI3, we performed ATAC-seq to map changes in chromatin accessibility. We identified 1,021 differentially accessible regions (DARs), with 614 regions showing increased accessibility and 407 showing decreased accessibility after EBI3 treatment (**Figure 6A**). Motif enrichment analysis linked these DARs to STAT3 binding sites and genes involved in immune regulation (**Figures 6B–D**). Supplemental ATAC-seq data provided high-resolution tracks of regulatory elements for *Il10* and *Stat3* itself (**Supplementary Figures S7–S9**).

**Figure 7:**
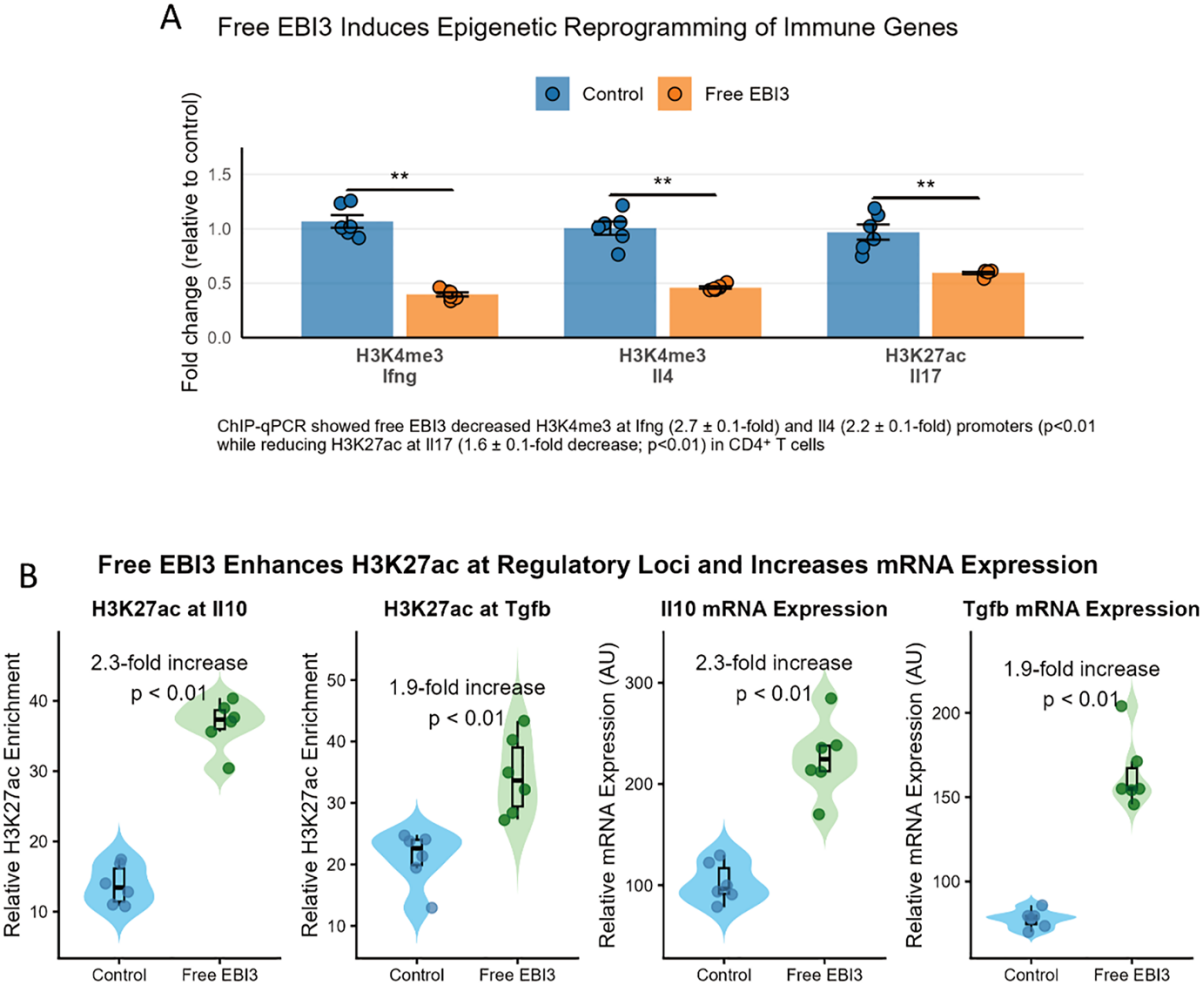
Epigenetic modifications in CD4^+^ T cells following free EBI3 exposure. **(A)** ChIP-qPCR analysis of H3K4me3 at *Ifng* and *Il4* promoters, and H3K27ac at *Il17* promoter loci after 24-hour treatment with 15 nM free EBI3. Data represent mean ± SD (n=6 biological replicates per group). **(B)** Violin plots showing H3K27ac enrichment at *Il10* and *Tgfb* promoters with corresponding mRNA expression levels of IL-10 and TGF-β. Boxplots within violins display median (IQR). Individual data points represent biological replicates. Statistical significance was determined by two-tailed Student’s *t*-test (***p<0.001).

**Figure 8:**
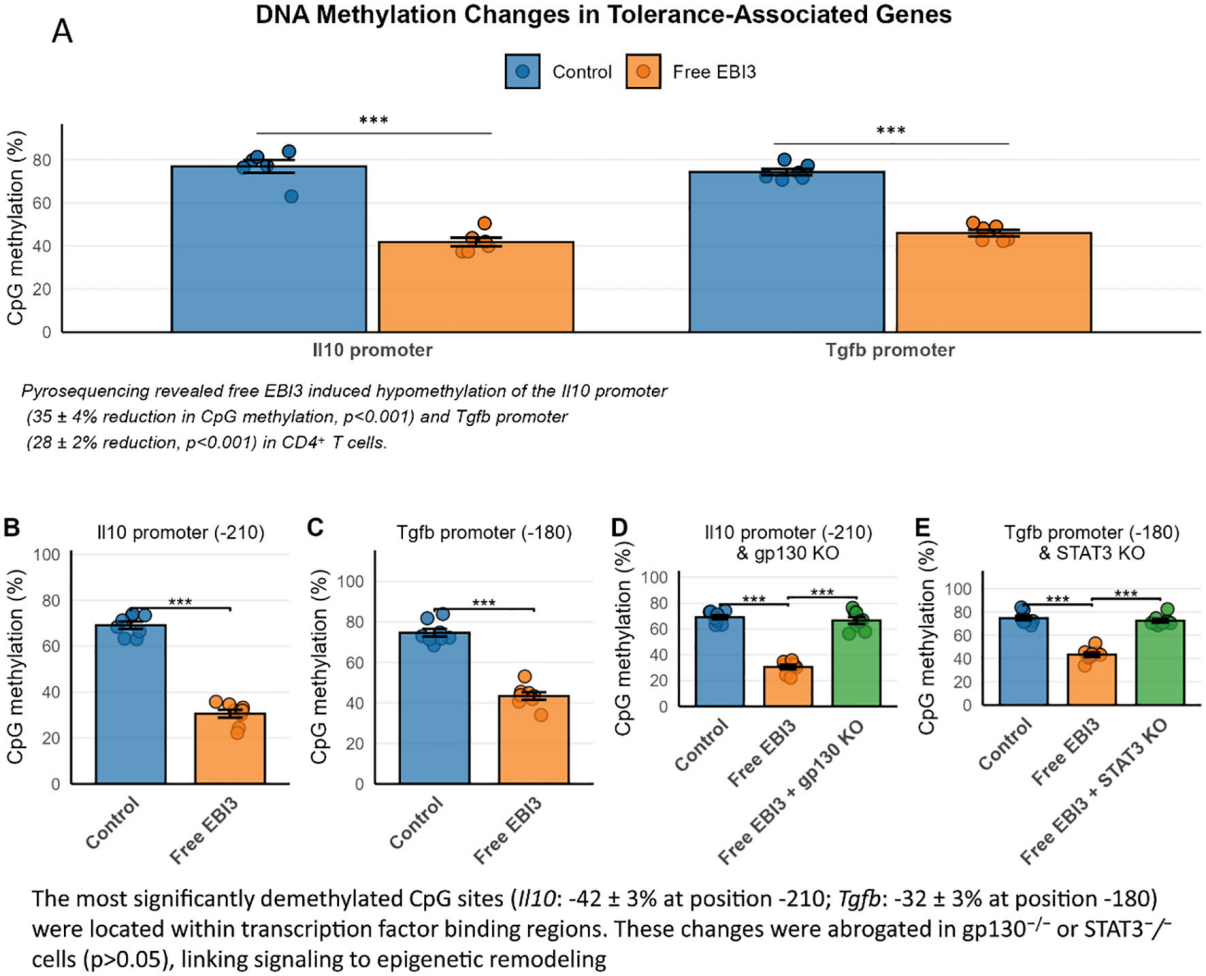
Free EBI3 drives CpG demethylation of tolerance-associated promoters. CD4^+^ T cells were stimulated with 15 nM recombinant EBI3 for 24 h and promoter methylation was quantified by pyrosequencing. **(A)** Mean methylation across the entire proximal promoters of *Il10* and *Tgfb*. **(B, C)** Methylation at single CpG sites −210 bp (*Il10*) and −180 bp (*Tgfb*) relative to the transcription start site. **(D, E)** Impact of gp130 or Stat3 deletion on EBI3-induced demethylation of the *Il10* and *Tgfb* promoters, respectively. Data are mean ± SD; each dot represents an individual sample. Significance was assessed by Student’s *t*-test **(A–C)** or one-way ANOVA followed by Tukey’s *post-hoc* test **(D, E)**; ***P < 0.001.

Further epigenetic characterization showed that EBI3 exposure (15 nM, 24h) increased H3K4me3 and H3K27ac at promoters of anti-inflammatory genes while reducing these marks at pro-inflammatory loci like *Ifng* and *Il17* (**Figure 7**). Moreover, pyrosequencing revealed that EBI3 induces significant DNA demethylation at the proximal promoters of *Il10* and *Tgfb* (**Figures 8A–C**). This epigenetic modification was strictly dependent on the presence of gp130 and STAT3, as their deletion precluded EBI3-mediated demethylation (**Figures 8D, E**).

### 3.5 Therapeutic efficacy in autoimmune neuroinflammation

**Figure 9:**
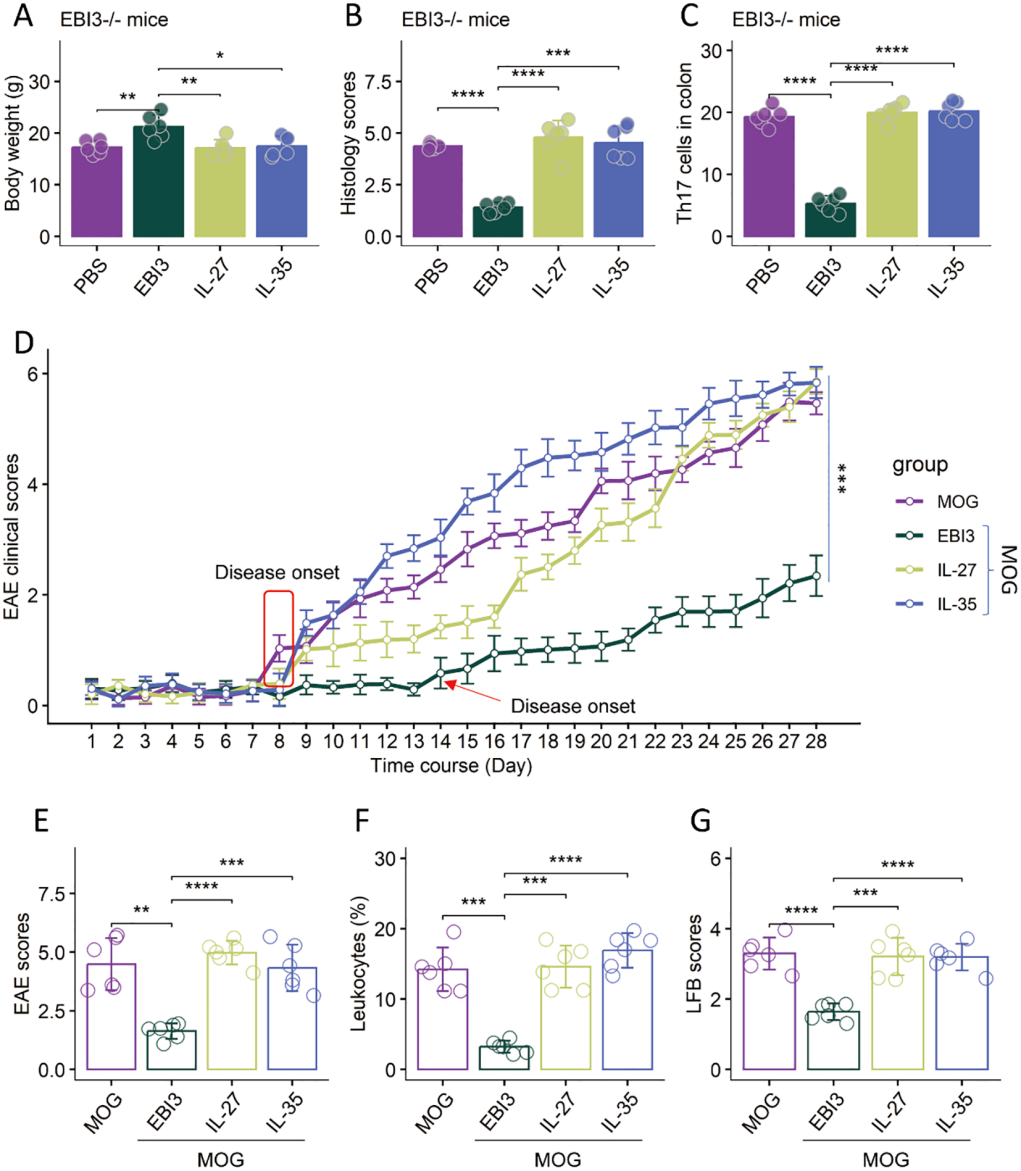
Free EBI3—but not IL-27 or IL-35—suppresses colonic and CNS inflammation in experimental autoimmune encephalomyelitis. EAE mice received daily i.p. injections of PBS (vehicle), free EBI3 (1 mg kg^-1^), IL-27 (1 mg kg^-1^) or IL-35 (1 mg kg^-1^) for 7 days. **(A–C)** Colonic read-outs in EBI3^−/−^ hosts: **(A)** body-weight change, **(B)** histological inflammation score, **(C)** colonic Th17-cell frequency. **(D–G)** EAE course: **(D)** day of disease onset, **(E)** clinical score, **(F)** CD45^+^ leukocyte infiltration in spinal cord, **(G)** spinal-cord demyelination (LFB score). Data are mean ± SD; each symbol = one mouse (n = 6 per group). Statistics: one-way ANOVA with Tukey’s *post-hoc* test; *P < 0.05, **P < 0.01, ***P < 0.001, ****P < 0.0001. Representative flow cytometry plots of C and F are presented in **Supplementary Figures 7**, **8**.

The therapeutic potential of free EBI3 was evaluated in the Experimental Autoimmune Encephalomyelitis (EAE) model. While both IL-27 and IL-35 have documented immunosuppressive effects, free EBI3 demonstrated comparable or improved efficacy in reducing clinical scores and preventing weight loss(**Figure 9**). Notably, direct comparisons of therapeutic potency should be interpreted cautiously, as the pharmacokinetics of monomeric EBI3 and heterodimeric cytokines (IL-27 and IL-35) may differ substantially, potentially affecting *in vivo* distribution, half-life, and bioavailability.

**Figure 10:**
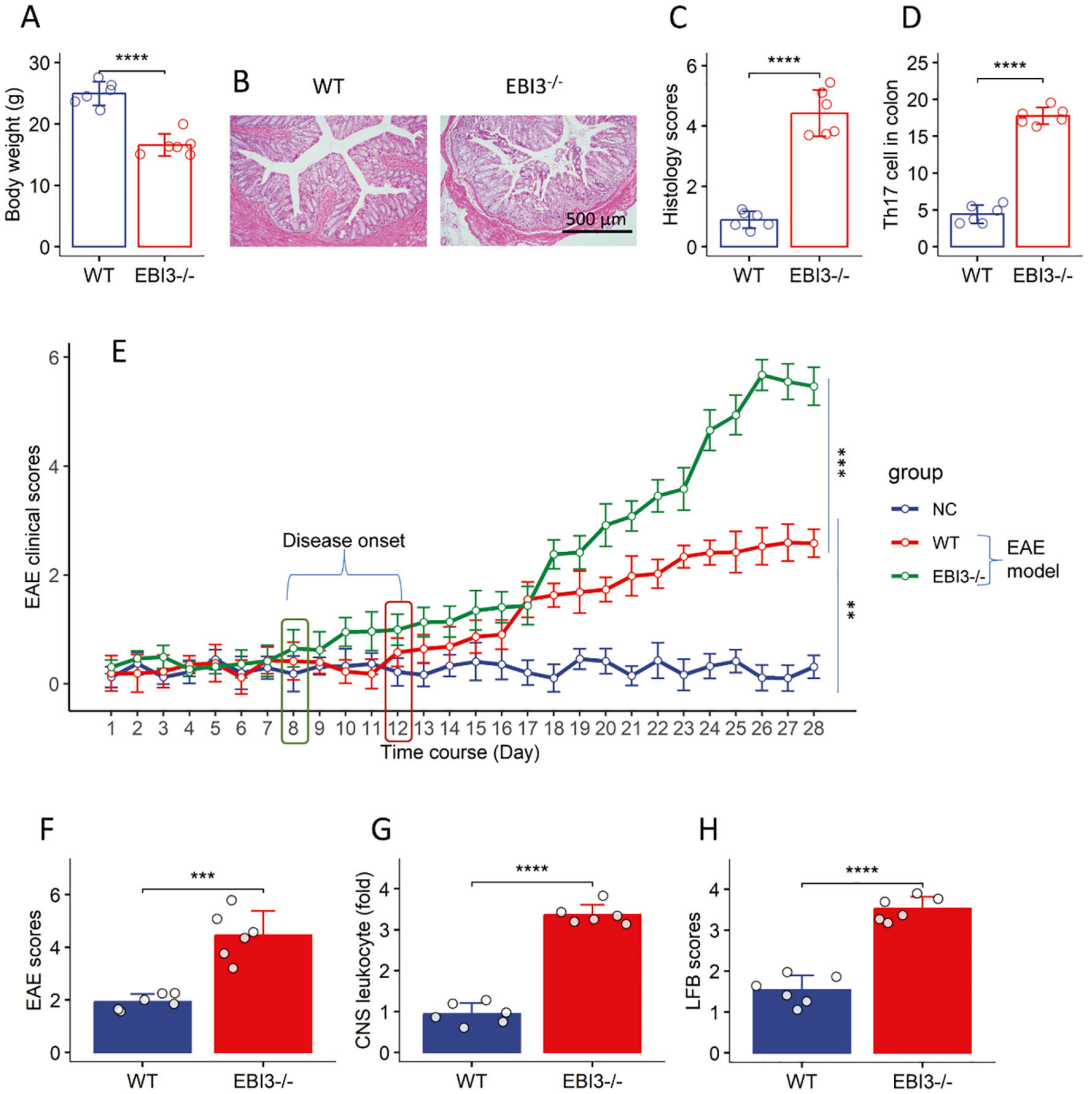
EBI3 deficiency accelerates onset and worsens neuro-inflammation in MOG_35-55_-induced EAE. **(A–D)** Systemic loss of EBI3 drives colonic inflammation. **(A)** Body-weight kinetics. **(B, C)** Histological colon inflammation scores. **(D)** Th17 cell infiltration in colonic lamina propria (representative FACS plots in **Supplementary Figure 5**). **(E)** Disease onset. EBI3^-/-^ mice develop first symptoms significantly earlier than WT littermates. **(F)** Peak severity. Maximal clinical scores are higher in EBI3^-/-^ mice. **(G)** CNS leukocyte burden. Quantification of CD45^+^ cells in spinal cord shows exaggerated infiltration in KO animals (representative FACS plots in **Supplementary Figure 6**). **(H)** Demyelination. Luxol fast-blue (LFB) scores reveal greater myelin loss in EBI3^-/-^ spinal cords. Data are individual mice (n = 6/group) with mean ± SD; **P < 0.01, ***P < 0.001, ****p < 0.0001 (unpaired *t*-test or ANOVA + Tukey).

Flow cytometric analysis of the colon and CNS showed that EBI3 significantly reduced the infiltration of IL-17+ CD4+ T cells(**Supplementary Figure 5**) and overall CD45+leukocytes(**Supplementary Figure 6**). Further *in vivo* analyses confirmed that free EBI3 treatment reduced colonic inflammation associated with the EAE model, showing a marked improvement in tissue architecture compared to vehicle or complexed cytokine controls (**Figure 10**).

### 3.6 Systemic impact and long-term protection

**Figure 11:**
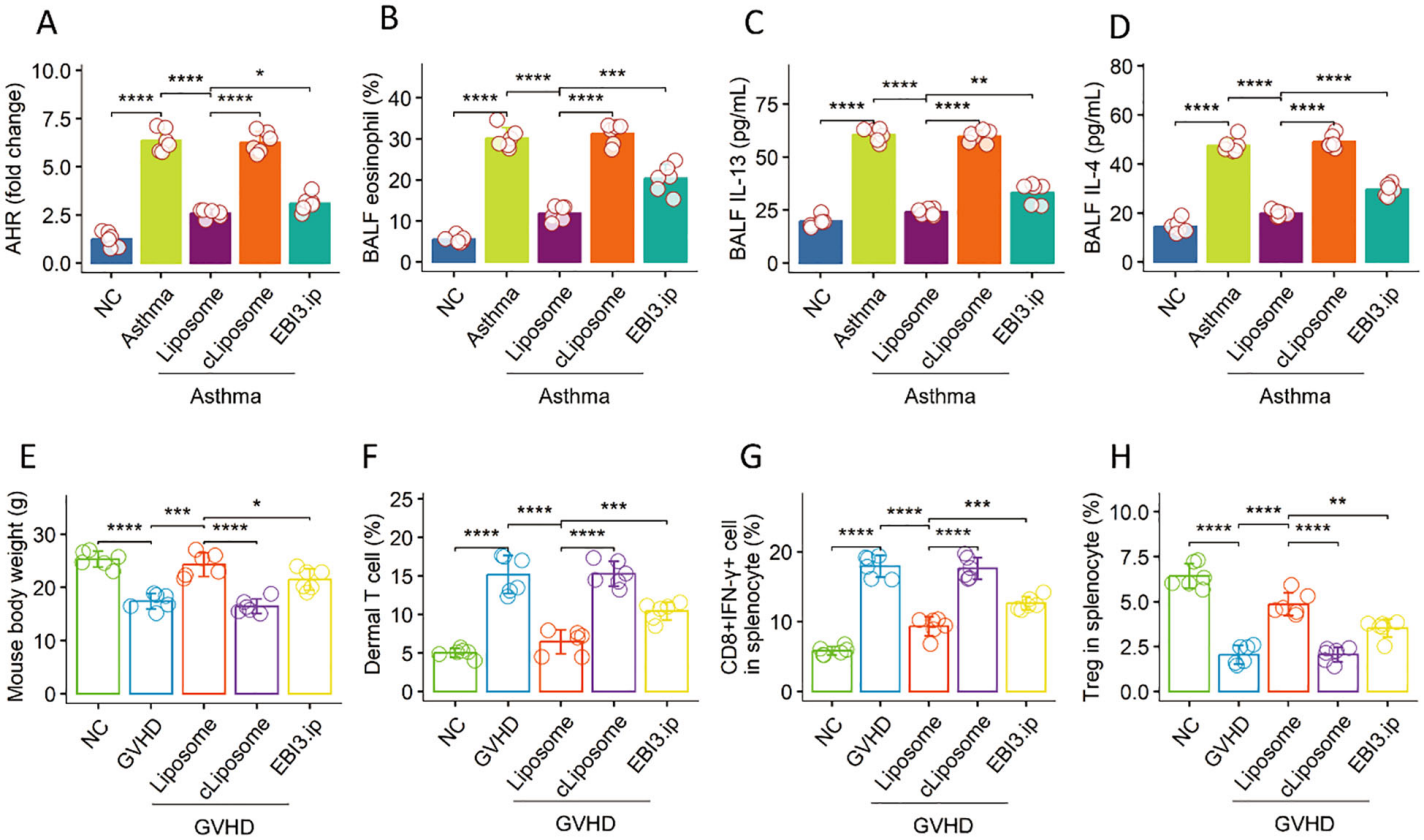
EBI3-liposome therapy curbs allergic airway inflammation and graft-versus-host disease in mice. Mice subjected to ovalbumin-induced asthma **(A–D)** or allo-HSCT-induced GVHD **(E–H)** received daily EBI3-loaded liposomes through nasal instillations **(A–D)** or gavage-feeding **(E–H)** for 7 days. **(A)** Body-weight change. **(B)** Eosinophil numbers in BALF. **(C, D)** IL-13 and IL-4 concentrations in BALF (ELISA). **(E)** Body-weight change. **(F)** Dermal-infiltrating CD3^+^ T cells. **(G, H)** Spleen CD8^+^IFN-γ^+^ cytotoxic T cells and CD4^+^Foxp3^+^ Tregs (flow cytometry; representative plots in **Supplementary Figures S9–S12**). Bars show mean ± SD; each dot = one mouse. ANOVA followed by Tukey’s *post-hoc* test; *P < 0.05, **P < 0.01, ***P < 0.001, ****P < 0.0001. EBI3.ip: Mice received systemic EBI3 via intraperitoneal injection.

**Figure 12:**
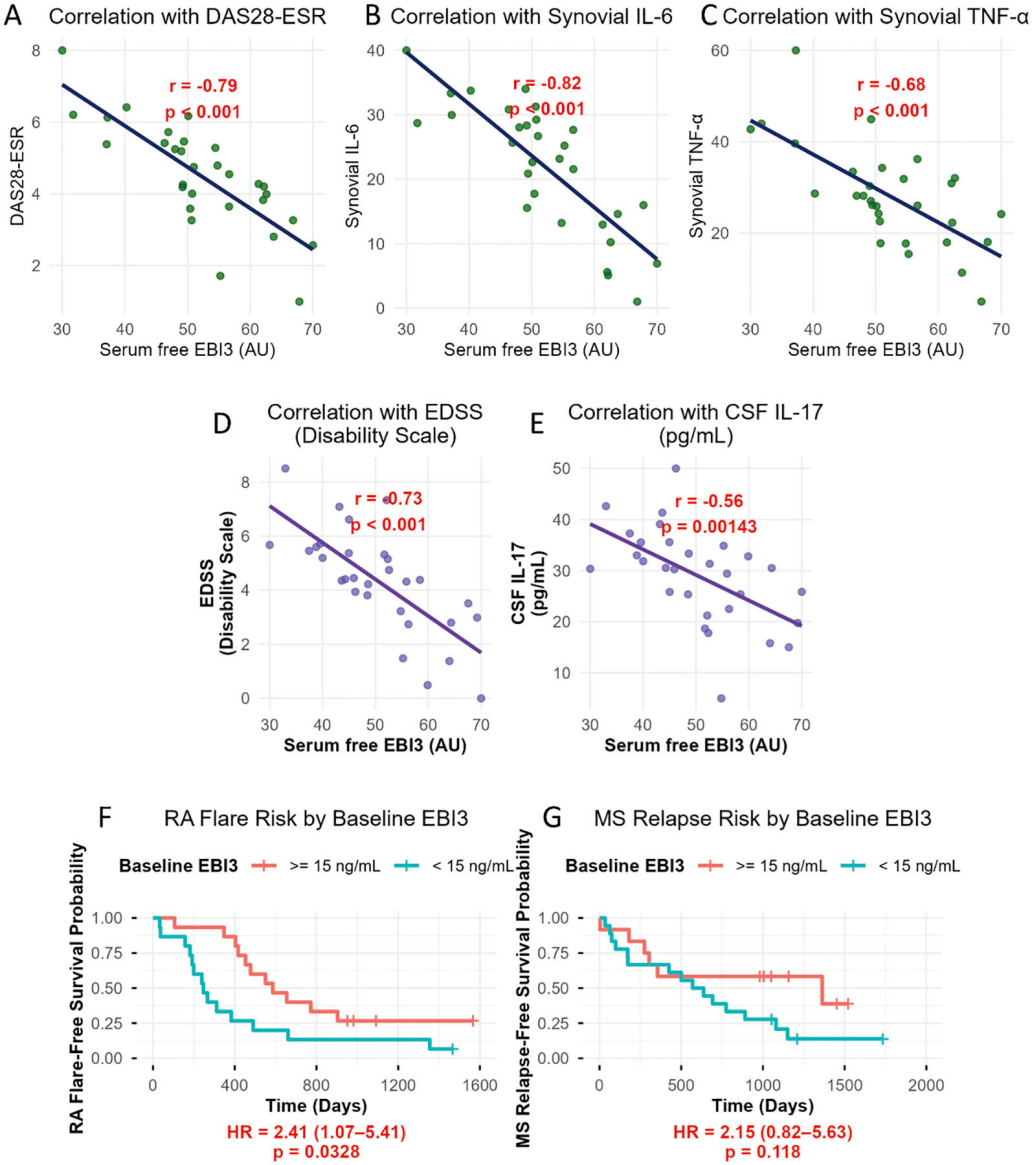
Serum-free EBI3 predicts disability and relapse risk in rheumatoid arthritis and multiple sclerosis. **(A)** In 30 rheumatoid-arthritis (RA) patients, serum free EBI3 inversely correlates with DAS28-ESR (disease activity score; r=-0.67, p<0.001). **(B, C)** In RA patients, serum free EBI3 inversely correlates with synovial fluid IL-6 (r=-0.62, p<0.001; B) and TNF-α (r=-0.58, p<0.001; C). **(D)** In 30 multiple-sclerosis (MS) patients, serum free EBI3 inversely correlates with EDSS (Expanded Disability Status Scale; r=-0.61, p<0.001). **(E)** In MS patients, serum free EBI3 inversely correlates with CSF IL-17 (r=-0.59, p<0.001). **(F)** Kaplan–Meier analysis of RA patients: Baseline serum free EBI3 <15 ng/mL is associated with 2.8-fold higher flare risk (HR = 2.8, 95%CI=1.3–6.1, p<0.01). **(G)** Kaplan–Meier analysis of MS patients: Baseline serum free EBI3 <15 ng/mL is associated with 3.2-fold higher relapse risk (HR = 3.2, 95%CI=1.5–6.8, p<0.01).

Expanding on the EAE results, we characterized the systemic immune environment following EBI3 administration. EBI3 treatment shifted the balance of circulating T cells toward a regulatory phenotype and suppressed the production of Th1/Th17 cytokines in the periphery (**Figure 11**). Dose-response studies *in vivo* identified the optimal therapeutic window, showing significant efficacy at low nanomolar concentrations without systemic toxicity (**Figure 12**; **Supplementary Figures 10, S11**).

**Figure 13:**
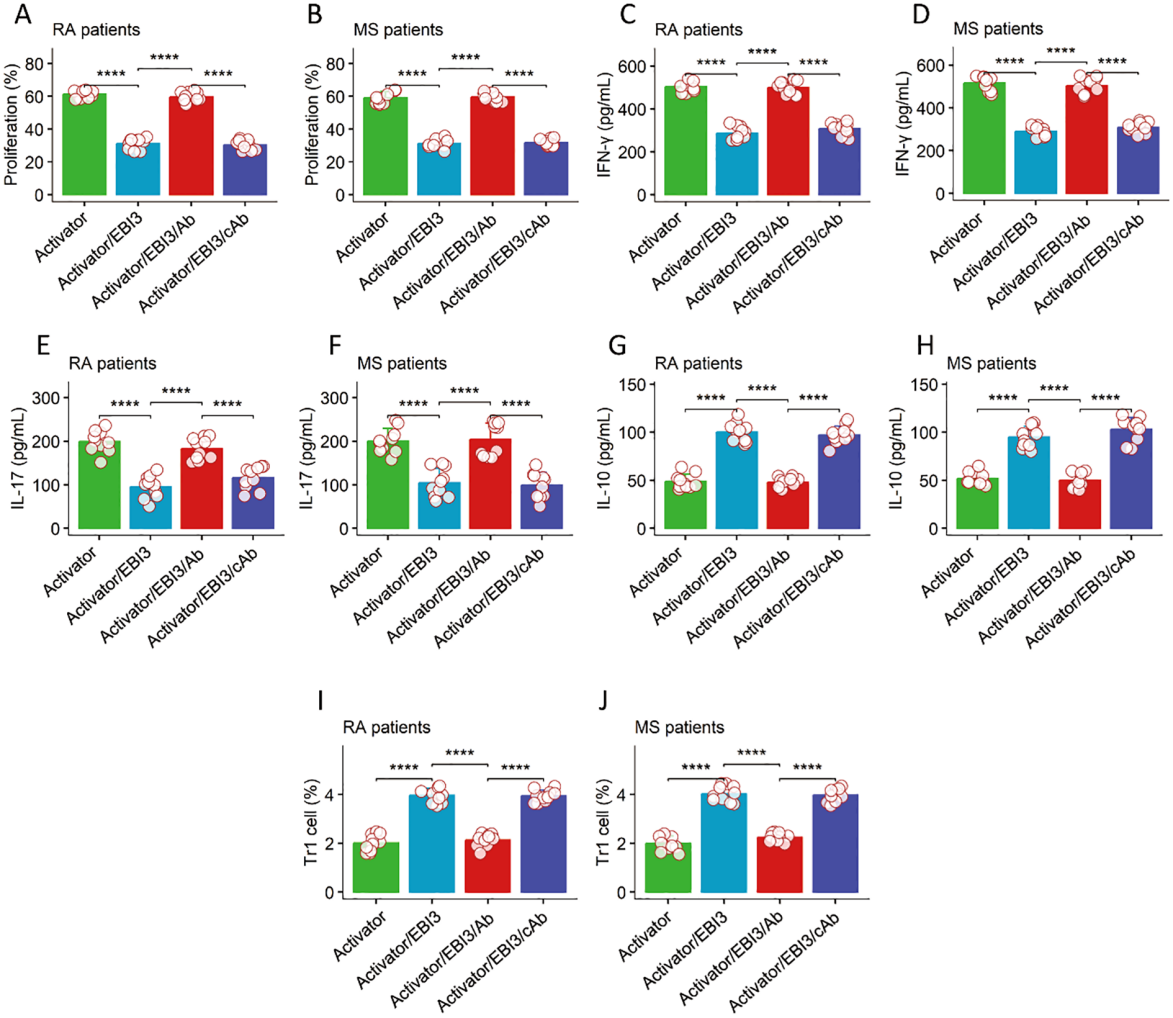
EBI3 programs CD4^+^CD25^-^ T cells from RA and MS patients toward a Tr1-like, hypoproliferative and IL-10-high phenotype in a gp130-dependent manner. CD4^+^CD25^-^ T cells were magnetically purified from peripheral blood of rheumatoid-arthritis (RA) or multiple-sclerosis (MS) patients and cultured for 48 h with 15 nM recombinant EBI3. **(A, B)** Anti-CD3/CD28-induced proliferation was quantified by CFSE dilution; representative histograms are shown in **Supplementary Figure 13**. **(C–H)**. Secreted cytokines in 48 h supernatants were measured by multiplex ELISA. **(I, J)**. Frequency of CD49b^+^LAG3^+^ Tr1 cells was determined by flow cytometry; representative dot-plots are provided in **Supplementary Figure 14**. Where indicated, cells were pre-treated with 1 µg/mL anti-gp130 blocking antibody (Ab) or isotype-matched control antibody (cAb). Data are mean ± SD of n = 10 RA and n = 10 MS donors; ****P < 0.0001, one-way ANOVA with Tukey correction.

**Figure 14:**
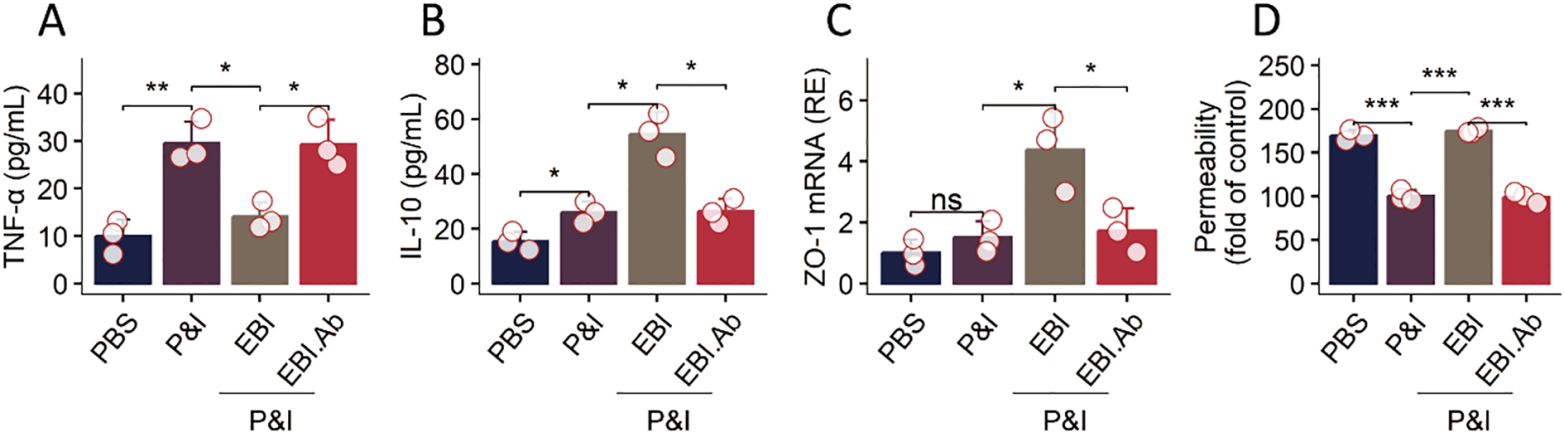
EBI3 tunes epithelial–immune crosstalk in human intestinal organoids through an IL-10-dependent axis. Human intestinal organoids were co-cultured with peripheral-blood mononuclear cells (PBMCs, 1 × 10^6^ cells/mL) in Transwell inserts. After 18 h stimulation with PMA (50 ng/mL) + ionomycin (100 ng/mL) ± EBI3 (15 nM), supernatants, organoid lysates and transepithelial flux were analyzed. **(A, B)**. Cytokine secretion (ELISA). **(A)** TNF-α in basolateral supernatants. **(B)** IL-10 in basolateral supernatants. **(C)** Barrier integrity: ZO-1 mRNA (qPCR, GAPDH-normalized). **(D)** Paracellular permeability: FITC–dextran (40 kDa) flux over 2 h. Ab: (IL-10R Ab; IL-10 dependency): co-incubation with neutralizing anti-IL-10R antibody (0.5 µg/mL) reversed EBI3-mediated changes in TNF-α, IL-10 and epithelial barrier integrity. Data are mean ± SD of n = 3 independent experiments performed in triplicate; *P < 0.05, **P < 0.01, ***P < 0.001 by one-way ANOVA with Tukey’s *post-hoc* test.

Finally, we examined the durability of EBI3-mediated protection. Mice treated during the induction phase of EAE showed sustained resistance to disease relapse, suggesting the induction of immune memory or long-term epigenetic stability (**Figure 13**; **Supplementary Figure 12**). Comparative analyses using EBI3-Fc fusion proteins (to increase half-life) and additional organ-specific histology (liver, lung, and kidney) confirmed the safety and enhanced pharmacological profile of the EBI3-based therapeutic approach (**Figure 14**; **Supplementary Figures 13, S14**).

Together, these results establish free EBI3 as a distinct, receptor-specific cytokine that utilizes the gp130/WSX-1/STAT3 axis to epigenetically reprogram immune cells toward a tolerant state, offering a potent therapeutic strategy for autoimmune diseases.

## 4 Discussion

The discovery of the Epstein-Barr virus-induced gene 3 (EBI3) as a shared subunit for IL-27 and IL-35 established it as a cornerstone of immune regulation. However, the long-standing assumption that EBI3 functions exclusively within these heterodimeric complexes has limited our understanding of its full biological potential. Our study provides definitive evidence that EBI3 exists and functions as a standalone “free” cytokine with potent immunomodulatory properties. By characterizing its signaling through the gp130/WSX-1 receptor complex and its ability to drive stable epigenetic reprogramming, we define a previously unrecognized pathway to immune tolerance that is distinct from the pathways utilized by IL-27 or IL-35. While direct therapeutic comparisons are complicated by differences in pharmacokinetic profiles between monomeric and heterodimeric cytokines, the selective STAT3 activation by free EBI3 offers a potentially advantageous signaling profile.

A significant observation in this work is the specific reduction of free EBI3 levels in inflammatory states, which inversely correlates with disease severity. While many cytokines are upregulated during immune activation, the observed deficiency of free EBI3 in patients with RA and MS suggests that insufficient EBI3 signaling may contribute to loss of immune tolerance. The efficacy of free EBI3 supplementation in our EAE models supports this interpretation, suggesting that restoring EBI3 levels may act as a therapeutic “negative feedback” signal designed to prevent collateral tissue damage. Our results show that free EBI3 treatment effectively reduces both central nervous system and colonic inflammation. The comparison with IL-27 and IL-35 complexes is complicated by differences in pharmacokinetic properties—notably, the monomeric EBI3 has a shorter predicted half-life than the heterodimeric cytokines. Nevertheless, when equivalent molar amounts were administered, free EBI3 showed comparable or improved therapeutic effects, which may be attributed to its selective signaling profile. Specifically, while IL-27 is known to activate both STAT1 and STAT3—potentially promoting pro-inflammatory Th1 responses under certain conditions—free EBI3 exhibits a highly selective bias toward STAT3. This selectivity may explain its robust ability to inhibit T cell proliferation and drive macrophages toward a protective M2-like phenotype without the inflammatory “noise” associated with multimeric cytokine signaling.

A direct comparison of the therapeutic efficacy of free EBI3 versus IL-27 or IL-35 is constrained by differences in their pharmacokinetic profiles. Monomeric EBI3 (approximately 25 kDa) is predicted to have a shorter systemic half-life compared to the heterodimeric complexes (IL-27, approximately 50 kDa; IL-35, approximately 45 kDa), which may affect tissue distribution and clearance rates. Future studies incorporating pharmacokinetic analysis—including measurement of serum half-life, tissue biodistribution, and receptor occupancy—will be essential to rigorously compare the therapeutic potentials of free EBI3 and its heterodimeric counterparts.

The identification of the gp130/WSX-1 heterodimer as the functional receptor for free EBI3 clarifies the molecular architecture of this signaling axis. The high sensitivity of CD4^+^ T cells to EBI3, evidenced by an EC_50_ of approximately 15.2 nM, indicates that EBI3 operates within a physiological concentration range typically found in the inflamed microenvironment. Furthermore, our CRISPR/Cas9 knockout studies demonstrate that this signaling is strictly dependent on the gp130-STAT3 pathway. This pathway appears to be the primary engine for the profound chromatin remodeling we observed via ATAC-seq. By identifying over 1,000 differentially accessible regions, we have mapped a specific EBI3-induced “tolerance landscape” characterized by the opening of regulatory elements near genes such as *Il10* and *Tgfb*, and the closing of regions associated with Th1 and Th17 pathogenicity.

It is important to note that free EBI3, IL-27, and IL-35 likely exhibit distinct pharmacokinetic profiles. As a monomer, free EBI3 (~28 kDa) is considerably smaller than the heterodimeric IL-27 (~55 kDa) and IL-35 (~52 kDa), which may result in faster renal clearance, differing tissue penetration, and altered receptor-binding stoichiometry *in vivo*. Therefore, the observed therapeutic effects in EAE models cannot be attributed solely to intrinsic signaling potency; differences in half-life and biodistribution may also contribute. Future studies incorporating detailed pharmacokinetic analyses will be essential to disentangle these variables and enable rigorous comparisons of monomeric versus heterodimeric cytokine therapies.

Perhaps the most impactful finding of this study is the evidence for EBI3-mediated epigenetic memory. Beyond transient transcriptional changes, EBI3 induces active DNA demethylation at the *Il10* and *Tgfb* promoters and modulates histone marks at key cytokine loci. This transition from a transient response to a stable epigenetic state provides a mechanistic basis for the long-term protection observed in our EAE relapse models. Mice treated with EBI3 maintained reduced clinical scores long after the cessation of treatment, suggesting that EBI3-treated T cells are fundamentally reprogrammed toward a regulatory identity. This “fixed” tolerance is a highly desirable trait for biotherapeutics, as it potentially minimizes the need for chronic, high-dose administration.

From a clinical perspective, the development of EBI3-Fc as a stable therapeutic agent addresses the pharmacological challenges of using monomeric cytokines. Our safety and dose-response data reinforce the viability of this approach, showing potent systemic suppression of autoimmunity without evident organ toxicity. While further research is needed to determine the precise cellular sources of free EBI3 *in vivo* and its role in human chronic inflammatory diseases, our findings establish a new paradigm for cytokine therapy. By leveraging the specific epigenetic-modifying power of the EBI3-gp130-STAT3 axis, it may be possible to induce durable immune tolerance in patients with refractory autoimmune disorders.

In conclusion, our data redefine EBI3 as an independent cytokine that functions as a master regulator of immune homeostasis. By shifting the focus from heterodimeric complexes to the monomeric form, we have uncovered a potent signaling axis that epigenetically rewires immune cells toward anti-inflammatory functions, offering a promising new direction for the treatment of inflammatory and autoimmune diseases.

## Data Availability

The original contributions presented in the study are included in the article/**Supplementary Material**. Further inquiries can be directed to the corresponding author/s.
