# Supplementary material for: Free Epstein-Barr virus-induced gene 3 functions as an autonomous immunosuppressive cytokine to maintain immune tolerance
Source: Front Immunol. 2026 Jul 27;17:1858675. doi: 10.3389/fimmu.2026.1858675 (PMC13453686; doi:10.3389/fimmu.2026.1858675)
Supplement: Supplementary file 1 [file DataSheet1.docx]

**Supplemental materials**

**Reagents**

Recombinant human EBI3 (ProSpec CYT-1088) and mouse EBI3 (Finetest Biotech ET1703-66) were produced in HEK293 cells, purified via Ni-NTA chromatography, and validated for purity (>95%) by SDS-PAGE (Coomassie staining) and LC-MS (sequence coverage: 98%). Endotoxin levels were quantified via LAL assay (Lonza PYROGENT-5000) and confirmed <0.1 EU/μg.

Recombinant human IL-27 (R&D 2526-IL) and IL-35 (R&D 8444-IL) were used as controls.

Neutralizing antibodies: Anti-human/mouse gp130 (R&D MAB228; 10 μg/mL), anti-human/mouse WSX-1 (R&D MAB1680; 10 μg/mL), anti-human/mouse p28 (R&D MAB1835; 5 μg/mL), anti-human/mouse p35 (R&D MAB446; 5 μg/mL), anti-human/mouse EBI3 (R&D MAB6664; 5 μg/mL). Isotype-matched IgG (R&D MAB002) served as a control.

**Cell Culture**

Naïve CD4⁺ T cells: Isolated from human peripheral blood (healthy controls/RA/MS patients) or C57BL/6 mouse spleens using the CD4⁺ T Cell Isolation Kit (Miltenyi 130-104-454) followed by CD45RA⁺ magnetic sorting (Miltenyi 130-094-531), yielding >98% purity (flow cytometry validation). Cultured in RPMI-1640 (Gibco 11875-093) supplemented with 10% heat-inactivated FBS (Gibco 10082-147), 1% penicillin-streptomycin (Gibco 15140-122), and 50 μM β-mercaptoethanol (Sigma M7522).

Bone marrow-derived dendritic cells (BMDCs): Isolated from C57BL/6 mouse femurs and tibias (flushed with RPMI-1640). Cultured in RPMI-1640 + 10% FBS + 20 ng/mL GM-CSF (PeproTech 315-03) for 7 days (media changed on days 3 and 5). Validated as CD11c⁺MHCII⁺ (>90% purity) via flow cytometry.

Bone marrow-derived macrophages (BMDMs): Isolated from C57BL/6 mouse femurs and tibias. Cultured in RPMI-1640 + 10% FBS + 20 ng/mL M-CSF (PeproTech 315-02) for 7 days (media changed on days 3 and 5). Validated as F4/80⁺CD11b⁺ (>90% purity) via flow cytometry.

Jurkat T cells (ATCC TIB-152) and RAW264.7 macrophages (ATCC TIB-71) were cultured in IMDM (Gibco 12440-053) + 10% FBS + 1% penicillin-streptomycin.

gp130⁻/⁻, WSX-1⁻/⁻, and STAT3⁻/⁻ Jurkat cells were generated via CRISPR/Cas9:

- sgRNAs targeting human GP130 (5′-GACCTGAACCGCATCTACGA-3′), WSX1 (5′-GGTGAGCTGGTGGTGCTGAA-3′), and STAT3 (5′-GCTGTTGGTGCTGGTGCTGA-3′) were cloned into pSpCas9(BB)-2A-Puro (Addgene 48139).

- Cells were transfected with Lipofectamine 3000 (Thermo Fisher L3000015) per manufacturer instructions.

- Transfected cells were selected with puromycin (1 μg/mL, Sigma P8833) for 7 days.

- Knockout efficiency (>90%) was confirmed by immunoblot (anti-gp130: Cell Signaling 3732; anti-WSX-1: Santa Cruz sc-514472; anti-STAT3: Cell Signaling 9139) and flow cytometry (surface gp130/WSX-1 detection, BioLegend 353402/356502).

**Macrophage Polarization**

BMDMs or RAW264.7 cells (2×10⁵/well) were seeded in 24-well plates and activated with LPS (100 ng/mL, Sigma L2630) for 2 h, then treated with free EBI3 (15 nM) for 24 h. Polarization was assessed via:

- Flow cytometry: CD206 (M2 marker, BioLegend 141706) and CD86 (M1 marker, BioLegend 105008) expression (gated on live F4/80⁺CD11b⁺ cells).

- RT-qPCR: Total RNA was extracted via RNeasy Mini Kit (Qiagen 74104), reverse-transcribed to cDNA (Thermo Fisher 4368814), and amplified using SYBR Green (Qiagen 204141) on a StepOnePlus Real-Time PCR System (Thermo Fisher). Primers: Arg1 (Qiagen QT00134288), iNOS (Qiagen QT00100275), GAPDH (Qiagen QT00199633). Results were normalized to GAPDH (ΔΔCt method).

**Dendritic Cell Maturation**

BMDCs (2×10⁵/well) were seeded in 24-well plates and stimulated with LPS (100 ng/mL) + free EBI3 (15 nM) for 24 h. Maturation was analyzed via:

- Flow cytometry: CD80 (BioLegend 104708), CD86 (BioLegend 105008), and MHCII (BioLegend 107622) expression (gated on live CD11c⁺ cells).

- IL-12p70 secretion: Quantified via Luminex (Millipore HCYTOMAG-60K) in cell supernatants.

**Signaling Pathway Analysis**

Cells (5×10⁵/well) were treated with free EBI3 (15 nM) for 0, 15, 30, or 60 min, then harvested and lysed in RIPA buffer (Thermo Fisher 89900) supplemented with protease/phosphatase inhibitor cocktail (Thermo Fisher 78440) for 30 min on ice. Lysates were centrifuged at 12,000 × g for 15 min at 4°C to collect supernatants.

Sandwich ELISA was used to detect phosphorylated/total proteins: pSTAT3 (Tyr705, Cell Signaling 9145), total STAT3 (Cell Signaling 9139), pSTAT1 (Tyr701, Cell Signaling 9167), total STAT1 (Cell Signaling 9172), pAKT (Ser473, Cell Signaling 4060), and total AKT (Cell Signaling 9272). Assays followed the manufacturer’s protocols, with absorbance read at 450 nm.

STAT3 nuclear translocation: Cells were fixed with 4% paraformaldehyde (Sigma P6148) for 15 min at RT, permeabilized with 0.1% Triton X-100 (Sigma T8787) for 10 min, and blocked with 5% BSA (Sigma A7906) for 1 h. Stained with anti-pSTAT3 (1:200, Cell Signaling 9145) overnight at 4°C, followed by Alexa Fluor 488-conjugated secondary antibody (1:500, Thermo Fisher A11001) for 1 h at RT. Nuclei were counterstained with DAPI (Thermo Fisher D1306, 1:1000). Images were acquired via Zeiss LSM 880 confocal microscope (63× oil objective). Nuclear/cytoplasmic pSTAT3 ratios were calculated using ImageJ (NIH) with the “Nuclear Pore” plugin.

**Epigenetic Analysis**

**Chromatin Immunoprecipitation (ChIP)-qPCR**

CD4⁺ T cells (1×10⁷) treated with free EBI3 (15 nM) for 24 h were cross-linked with 1% formaldehyde (Sigma F8775) for 10 min at RT, quenched with 0.125 M glycine for 5 min, and lysed in ChIP lysis buffer (Cell Signaling 9002) with inhibitors. Chromatin was sonicated using a Bioruptor Pico (Diagenode) to generate 200–500 bp fragments (30s on/30s off, 10 cycles, 4°C).

Immunoprecipitation was performed with 5 μg of antibody: H3K4me3 (Abcam ab8580), H3K27ac (Abcam ab4729), or IgG control (Abcam ab172730) overnight at 4°C. Protein A/G magnetic beads (Thermo Fisher 88802) were added for 2 h at 4°C. Beads were washed with ChIP wash buffers (Cell Signaling 9003), and DNA was eluted and purified via ChIP DNA Clean & Concentrator Kit (Zymo D5205).

qPCR was performed with primers targeting Ifng, Il4, Il17, Il10, and Tgfb promoters (sequences in Table S1) using SYBR Green (Qiagen 204141). Results were normalized to input DNA (ΔCt method).

**DNA Methylation Analysis**

Genomic DNA from CD4⁺ T cells was extracted via DNeasy Blood & Tissue Kit (Qiagen 69504), bisulfite-converted using EZ DNA Methylation-Gold Kit (Zymo D5005) per manufacturer instructions, and eluted in 20 μL elution buffer.

Pyrosequencing was performed on a Qiagen PyroMark Q24 using PyroMark Gold Q24 Reagents (979004). Primers targeted CpG sites in the Il10 and Tgfb promoters (Table S1). Methylation percentage per CpG site was calculated using PyroMark Q24 Software (v2.4).

**Assay for Transposase-Accessible Chromatin (ATAC)-seq**

CD4⁺ T cells (5×10⁴) treated with free EBI3 (15 nM) for 24 h were processed using the Nextera DNA Flex ATAC-seq Kit (Illumina 20018105) per the manufacturer’s protocol. Libraries were quantified via Qubit dsDNA HS Assay (Thermo Fisher Q32854) and sequenced on an Illumina NovaSeq 6000 (150 bp paired-end reads, 20 million reads per sample).

Bioinformatics analysis: Reads were aligned to the mm10 (mouse) or hg38 (human) genome using Bowtie2 (v2.4.4) with parameters “--local --very-sensitive-local --no-unal --no-mixed --no-discordant --phred33 -I 10 -X 700.” Duplicate reads were removed with Picard (v2.27.5). Peaks were called using MACS2 (v2.2.7.1) with “-q 0.05 --keep-dup all.” Differential accessibility was analyzed using DESeq2 (v1.38.3) in R; peaks with log₂(fold change) >1 and adj. p<0.05 were considered differentially accessible regions (DARs). Motif enrichment analysis was performed with HOMER (v4.11).

**Animal Models**

All animal experiments were approved by the Institutional Animal Care and Use Committee (IACUC 2023-004). Mice were housed in a specific pathogen-free (SPF) facility with 12 h light/dark cycles, ad libitum food, and water.

EBI3⁻/⁻ mice: C.129X1(B6)-Ebi3<sup>tm1Rsb</sup>/J mice (Jackson 005646) and wild-type (WT) C57BL/6 mice (Jackson 000664) were used at 8–10 weeks of age. Spontaneous colitis was monitored for 12 weeks: body weight (weekly), stool consistency (1=normal, 2=loose, 3=diarrhea), and occult blood (Hemoccult II, Beckman Coulter). Colonic tissue was fixed in 10% neutral buffered formalin (Sigma HT501128) for H&E staining (histology score: 0–4, based on inflammation, crypt loss, and ulceration) or digested with collagenase IV (Sigma C5138) for flow cytometry (IL-17⁺CD4⁺ T cells).

EAE model: Mice were immunized subcutaneously (s.c.) at the base of the tail with 200 μg MOG₃₅₋₅₅ peptide (Sigma M2158) emulsified in complete Freund’s adjuvant (CFA, Sigma F5881) + 500 ng heat-killed Mycobacterium tuberculosis (H37Ra, BD 231141). Pertussis toxin (Sigma P7208, 200 ng/mouse) was injected intraperitoneally (i.p.) on day 0 and 2. Clinical scores were recorded daily: 0=no symptoms; 1=tail limpness; 2=hind limb weakness; 3=hind limb paralysis; 4=forelimb paralysis; 5=death. CNS tissues (spinal cord, brain) were collected on day 21, homogenized, and processed via 30/70% Percoll gradient (GE Healthcare 17-0891-01) to isolate leukocytes for flow cytometry.

OVA-induced asthma: Mice were sensitized i.p. with 10 μg OVA (Sigma A5503) + 2 mg alum (Thermo Fisher 77161) on day 0 and 7. From day 14–16, mice were challenged with 1% OVA aerosol (30 min/day) using a Pari LC Sprint nebulizer (Pari Medical). Airway hyperresponsiveness (AHR) was measured 24 h after the last challenge using a flexiVent® FX (Scireq) with increasing doses of methacholine (0–50 mg/mL). Bronchoalveolar lavage fluid (BALF) was collected via tracheal cannulation, and eosinophils were counted via hemocytometer (gated on live CD45⁺Siglec-F⁺ cells). Lung tissue was fixed for histology or processed for RT-qPCR (IL-4/IL-13 expression).

GVHD model: BALB/c donor splenocytes (1×10⁷/mouse) were injected i.v. into irradiated (6 Gy, Cs-137 irradiator, J.L. Shepherd) C57BL/6 recipient mice. Mice were treated with free EBI3 (1 mg/kg/day, i.p.) or PBS from day 0–7. Clinical parameters: body weight (every 2 days), GVHD score (0=no symptoms; 1=ruffled fur; 2=diarrhea; 3=skin lesions; 4=severe wasting), and dermal T cell infiltration (H&E staining of ear skin).

EBI3⁻/⁻ mice with colitis (week 10) or EAE (day 8 post-immunization) were treated with: (a) free EBI3 (1 mg/kg/day, i.p.), (b) IL-27 (1 mg/kg/day, i.p.), (c) IL-35 (1 mg/kg/day, i.p.), or (d) PBS for 7 days. Outcomes: clinical scores, colonic/CNS leukocyte infiltration (flow cytometry), and cytokine levels (Luminex).

**Clinical Studies**

This study was approved by the Institutional Review Board (IRB 2022-0123). All participants provided written informed consent.

- RA patients: 50 patients meeting ACR/EULAR 2010 criteria, no biologic therapy in the past 3 months. Disease activity was assessed via DAS28-ESR.

- MS patients: 50 patients meeting McDonald 2017 criteria (relapsing-remitting MS, RRMS). Disability was assessed via EDSS.

- Healthy controls (HC): 30 individuals with no history of autoimmune/allergic diseases, matched to patients for age (±5 years), sex, and comorbidities (hypertension, diabetes).

*Note: Initial enrollment included 45 RA and 38 MS patients; 5 additional patients per group were enrolled to account for dropouts (final n=50 each).*

Samples were collected at baseline and 6-month follow-up. Prospective follow-up for RA flares (DAS28-ESR increase ≥1.2) and MS relapses (new MRI lesions/clinical symptoms) lasted 12 months post-baseline.

- Serum: Collected via venous puncture, centrifuged at 3000 × g for 10 min, and stored at -80°C. Free EBI3 levels were measured via custom ELISA.

- PBMCs: Isolated via Ficoll-Paque Plus (GE Healthcare 17-1440-02) density gradient centrifugation, cryopreserved in 10% DMSO + 90% FBS, and stored in liquid nitrogen.

- Synovial fluid (RA): Collected from 20 patients via arthrocentesis; CSF (MS): collected from 20 patients via lumbar puncture. Samples were centrifuged at 3000 × g for 10 min and stored at -80°C.

PBMC ex vivo rescue assays: Thawed PBMCs (1×10⁶/mL) were stimulated with anti-CD3/CD28 (1 μg/mL each, BioLegend 100314/102116) + free EBI3 (15 nM) for 72 h. Outcomes: T cell proliferation (CFSE), cytokine secretion (IL-6/TNF-α/IL-17/IL-10 via Luminex), and Tr1 cell frequency (CD4⁺CD49b⁺LAG3⁺ via flow cytometry).

### **Experimental Procedures for Figure 5C–D**

**(Jurkat cell proliferation inhibition assay in wild-type and gp130/WSX-1 knockout cells)**

Wild-type (WT) Jurkat cells and CRISPR/Cas9-generated **gp130⁻/⁻** or **WSX-1⁻/⁻** Jurkat cells were cultured in IMDM supplemented with 10% fetal bovine serum (FBS), 100 U/mL penicillin, and 100 μg/mL streptomycin at 37 °C in 5% CO₂. For proliferation assays, cells were washed twice with phosphate-buffered saline (PBS) and labeled with 5 μM carboxyfluorescein succinimidyl ester (CFSE; Thermo Fisher, C34554) for 15 min at 37 °C. The labeling reaction was quenched by adding 5 volumes of ice-cold complete IMDM, and cells were washed twice to remove unbound CFSE.

Labeled cells were seeded at a density of **1 × 10⁵ cells/well** in 96-well flat-bottom plates. Cells were stimulated with **anti-CD3/anti-CD28 activator beads** (1:1 bead-to-cell ratio; Thermo Fisher, 11161D) to induce proliferation, with or without recombinant free EBI3 (15 nM, Finetest Biotech, ET1703-66). The plates were incubated for 72 h at 37 °C, 5% CO₂.

After incubation, cells were harvested, washed once with cold PBS, and fixed in 1% paraformaldehyde for 15 min at room temperature. CFSE dilution (a marker of cell division) was analyzed by flow cytometry (BD FACSCymphony™ A5), gating on live single cells. Proliferation inhibition was calculated as:

**Proliferation inhibition (%) = [1 − (EBI3-treated proliferation index / Activator-only proliferation index)] × 100**

The proliferation index was defined as the percentage of cells that had undergone ≥1 division. All assays were performed in **3 independent biological replicates**, with technical duplicates per replicate. Data are presented as mean ± SD.

**Validation of CRISPR/Cas9 knockout efficiency in Jurkat cells**

Wild-type (WT) Jurkat cells and CRISPR/Cas9-edited gp130⁻/⁻, WSX-1⁻/⁻, and STAT3⁻/⁻ Jurkat cells were validated for knockout efficiency at both mRNA and protein levels.

For mRNA quantification, total RNA was extracted using RNeasy Mini Kit (Qiagen), reverse-transcribed, and analyzed by RT-qPCR with gene-specific primers (Table S1). Relative expression was normalized to GAPDH.

For protein validation, cell lysates were analyzed by Western blot using anti-gp130 (CST #3732), anti-WSX-1 (Santa Cruz sc-514472), and anti-STAT3 (CST #9139) antibodies. Surface expression of gp130 and WSX-1 was further confirmed by flow cytometry (BioLegend #353402, #356502).

Results showed >90% reduction of target mRNA and protein in each knockout cell line compared to WT Jurkat cells (Figure S3), confirming high knockout efficiency and specificity.

**Figure S1. EBI3 suppresses mouse CD4^+^ T cell proliferation**. Naive CD4⁺ T cells (CD4⁺CD62L^hi^), isolated from mouse spleens and labeled with CFSE, were cultured under the indicated conditions (above each subpanel) for 3 days. Gated flow cytometry histograms depict the proliferation profile of CD4⁺ T cells. Quantitative analysis of CD4⁺ T cell proliferation is provided in Figure 2C of the main text. Activator: Anti-CD3/CD28 antibody.

**Figure S2. EBI3 suppresses human CD4^+^ T cell proliferation**. Naive CD4⁺ T cells, isolated from healthy human peripheral blood mononuclear cells and labeled with CFSE, were cultured under the indicated conditions (above each subpanel) for 3 days. Gated flow cytometry histograms depict the proliferation profile of CD4⁺ T cells. Quantitative analysis of CD4⁺ T cell proliferation is provided in Figure 2D of the main text.

**Figure S3. CRISPR/Cas9-Mediated Knockout of gp130, WSX-1 and STAT3 Expression in Jurkat Cells.**
Jurkat cells (ATCC) were subjected to CRISPR/Cas9-mediated gene editing using specific guide RNAs targeting gp130 or WSX-1 or STAT3, or a non-targeting control CRISPR (cCRISPR) reagent (Santa Cruz Biotech), following the manufacturer’s protocol. Bar graphs show the **remaining mRNA expression levels of gp130, WSX-1 and STAT3** after knockout (by RT-qPCR). Data are represented as mean ± SD. Each individual data point (dot) corresponds to a single biological replicate. Statistical significance was determined by one-way ANOVA with Tukey's post-hoc multiple comparison test, comparing knockout cells (gp130⁻^/^⁻ or WSX-1⁻^/^⁻ or STAT3⁻^/^⁻) to control CRISPR (cCRISPR) treated cells. ****p*<0.05, ***p*<0.01.**

**Figure S4. The EBI3/gp130/WSX-1 axis regulates T cell proliferation.**

CFSE-labeled Jurkat T cells were cultured for three days under various conditions (indicated above each subpanel). Cell proliferation was subsequently analyzed by flow cytometry, with gated plots illustrating proliferating cells. KO denotes knockout. Jurkat cells with gp130 KO or WSX-1 KO or STAT3 KO refer to the knockout models described in Figure S3. Quantitative data for Jurkat cell proliferation are presented in Figure 5C-D of the main text. Activator: Anti-CD3/CD28 antibodies.

**Figure S5.** **Flow-cytometric analysis of colonic CD4⁺ IL-17⁺ T cells in EBI3-manipulated mice.**
A, Live singlets were first gated by exclusion of dead cells.
B, CD3⁺ CD4⁺ T cells were then selected and IL-17⁺ cells (C) were identified within this gate.
Absolute numbers of IL-17⁺ CD4⁺ T cells are reported in Fig. 9C. ViD: Viability staining dye.

**Figure S6. Quantification of leukocyte infiltration in the CNS.**

Single-cell suspensions prepared from mouse spinal cord were analyzed by flow cytometry. Representative pseudocolour plots show the gating strategy used to identify CD45^+^ leukocytes within the live-cell gate. Corresponding quantitative data are reported in Fig. 9G.

**Figure S7.** **Gating strategy and representative plots of colonic CD4⁺IL-17⁺ T cells in EBI3⁻^/^⁻ mice after cytokine treatment**
EBI3⁻^/^⁻ mice received daily i.p. injections for 7 days of: PBS (vehicle), free EBI3 (1 mg kg⁻¹), IL-27 (1 mg kg⁻¹), or IL-35 (1 mg kg⁻¹).
(A) Live singlets were gated by exclusion of dead cells (ViD⁻).
(B) CD3⁺CD4⁺ T cells were selected from live singlets.
(C) IL-17⁺ cells were identified within the CD3⁺CD4⁺ gate.
Absolute numbers of colonic CD4⁺IL-17⁺ T cells are quantified in Fig. 10C.

**Figure S8.** **Gating strategy and representative plots of CD45⁺ leukocytes in spinal cord of mice with EAE after cytokine treatment**
EAE mice received daily i.p. injections for 7 days of: PBS (vehicle), free EBI3 (1 mg kg⁻¹), IL-27 (1 mg kg⁻¹), or IL-35 (1 mg kg⁻¹).
(A) Live singlets were gated by exclusion of dead cells (ViD⁻).
(B) CD45⁺ cells were identified.
Absolute numbers of colonic leukocytes are quantified in Fig. 10F.

**Figure S9. Gating strategy for eosinophils in mouse BALF**. (A) Live cells were selected by exclusion of ViD^+^ dead cells. (B) Eosinophils were identified as EPX^+^ events within the live-cell gate. Corresponding quantification is shown in Fig. 11B. ViD, viability dye; NC, naïve control; Liposome, EBI3-loaded liposomes; cLiposome, empty liposomes. EBI3.ip: EBI3 was administered to mice via ip.

**Figure S10. Flow-cytometric quantification of skin-infiltrating T cells in a murine GVHD model.**
Single-cell suspensions generated from affected skin were sequentially gated on live leukocytes (A) and CD3^+^ T lymphocytes (B); absolute T-cell numbers are reported in Fig. 11F. Liposome: Liposomes were administered to mice via gavage-feeding. cLiposome: Empty liposome. EBI3.ip: EBI3 was administered to mice via ip.

**Figure S11. Assessment of CD8⁺IFN-γ⁺ T cells in the spleens of mice with graft-versus-host disease (GVHD).** Single-cell suspensions prepared from the spleens of GVHD-affected mice were sequentially gated on live leukocytes (Panel A), CD3⁺ T lymphocytes (Panel B), and CD8⁺IFN-γ⁺ T cells (Panel C). Absolute numbers of CD8⁺IFN-γ⁺ T cells are reported in Figure 11G. Liposome: Liposomes were administered to mice via gavage-feeding. cLiposome: Empty liposome. EBI3.ip: EBI3 was administered to mice via ip.

**Figure S12. Assessment of type 1 regulatory T cells (Tr1 cells) in the spleens of mice with graft-versus-host disease (GVHD).** Single-cell suspensions prepared from the spleens of GVHD-affected mice were sequentially gated on live splenocytes (A), CD3⁺CD4⁺ T lymphocytes (B), and CD49b^+^LAG3⁺ Tr1 cells (C). Absolute Treg numbers are reported in Fig. 11H. Liposome: Liposomes were administered to mice via gavage-feeding. cLiposome: Empty liposome. EBI3.ip: EBI3 was administered to mice via ip.

**Figure S13. EBI3 inhibits T-cell proliferation in RA and MS**. CFSE-labeled CD4^+^CD25⁻ T cells from 10 RA and 10 MS patients were stimulated with anti-CD3/CD28 ± 15 nM EBI3; proliferation was assessed by flow cytometry. Histograms (gated on CD4⁺ cells) show representative CFSE dilution. gp130 Ab: gp130 antibody (1 µg/mL). Isotype IgG was used as a negative control Ab. The quantitative data are presented in Fig. 13A-B.

**Figure S14. EBI3 restores Tr1 cells in patients with RA or MS**. PBMCs were obtained from RA patients (n = 10) or MS patients (n = 10). A, dead cells were gated out. B, CD3^+^CD4^+^ T cells were gated, from which CD49b^+^LAG3^+^ Tr1 cells were gated (C). The quantitative Tr1 cell data are presented in Fig. 13I-J.

Table S1: Primers Targeting Mouse Cytokine Promoters for ChIP-qPCR

| **Target Gene** | **Promoter Region (Relative to TSS)** | **Forward Primer Sequence (5′→3′)** | **Reverse Primer Sequence (5′→3′)** |
| --- | --- | --- | --- |
| Ifng | -180 to +20 bp | CTGCTGCTGAAGGTGGTGTA | GAGGAGGGAAGAGAAGAGGC |
| Il4 | -160 to +15 bp | CCAGAGTCCTGAGCCCTTTA | GCGGAGTTGGTGGTGATGTC |
| Il17 | -550 to -420 bp | GCAACAATTCCTGGCGTTAG | GGTTCATGTCATGGATGGTG |
| Il10 | -140 to +10 bp | CGGCTGCTGCTCTACATTTT | GGAGGGAGGGAAGAGAAGAG |
| Tgfb1 | -150 to +20 bp (Proximal) | CTGCTGCTCTACATTTTCGG | GGAGGGAAGAGAAGAGCGGT |
| Tgfb1 | -650 to -500 bp (Distal) | CCAGAGTCCTGAGCCCTTAC | GCGGAGTTGGTGGTGATGTA |
